# The impact of single-nucleotide variants of hepatitis B virus and antiviral on liver cancer in gray zone patients

**DOI:** 10.1186/s12929-025-01195-x

**Published:** 2025-12-01

**Authors:** Wei Teng, Ting-Tsung Chang, Chien-Wei Su, Jun-Hao Xu, Chiu-Chi Hsu, Yu-Cheng Chang, Wen-Chun Liu, Yu-Wei Chiou, Yu-Chuan Chang, Yuh-Jin Liang, Jaw-Ching Wu

**Affiliations:** 1 Institute of Clinical Medicine, National Yang Ming Chiao Tung University, Taipei, Taiwan, ROC; 2 Department of Gastroenterology & Hepatology, Chang Gung Memorial Hospital, Linkou Medical Center, Taoyuan, Taiwan, ROC; 3 Department of Internal Medicine, National Cheng Kung University Hospital, College of Medicine, National Cheng Kung University, Tainan, Taiwan, ROC; 4 Division of General Medicine, Department of Medicine, Taipei Veterans General Hospital, Taipei, Taiwan, ROC; 5 School of Medicine, College of Medicine, National Yang Ming Chiao Tung University, Taipei, Taiwan, ROC; 6 Department of Nursing, National Tainan Junior College of Nursing, Tainan, Taiwan, ROC; 7 Medical Research Department, Taipei Veterans General Hospital, Taipei, Taiwan, ROC; 8 Cancer and Immunology Center, National Yang Ming Chiao Tung University, Taipei, Taiwan, ROC

**Keywords:** Single-nucleotide variants, Pre-S/S region, Mitochondrial dysfunction, Treatment guidelines, Antiviral therapy

## Abstract

**Objectives:**

This study investigated the impact and related mechanisms of single-nucleotide variants (SNVs) in the HBV pre-S/S region on tumor development, and evaluated the role of antiviral therapy.

**Methods:**

A retrospective analysis was conducted in 104 patients of the gray zone. HCC-associated SNVs were analyzed in baseline samples.

**Results:**

HCC occurred in 15 patients (14.4%) during the median follow-up period of 10.4 years. Genotype B HBV-infected HCC patients had more T53C, A273G, and A529G SNVs and genotype C HBV-infected HCC patients had more T53C, G633A, and A3120G SNVs than HCC-free groups. Antiviral therapy reduced the risk of HCC in patients with HCC-associated SNVs in the gray zone both genotype B or C. Ectopic expression of replication-competent HBV plasmids in Huh7 cells expressing HCC-associated SNVs resulted in greater impairment of mitochondrial dynamics, increased production of reactive oxygen species (ROS), decreased mitochondrial membrane potential, lower ATP production, higher basal calcium levels, and reduced calcium buffering capacity compared to controls or wild-type HBV-expressing cells.

**Conclusions:**

CHB patients in the gray zone remain at risk for HCC owing to both wild-type and HCC-associated HBV SNVs, especially the latter, inducing mitochondrial and metabolic dysfunctions. Antiviral therapy reduces the risk of HCC development in these patients.

**Supplementary Information:**

The online version contains supplementary material available at 10.1186/s12929-025-01195-x.

## Introduction

Globally, over 240 million patients with chronic hepatitis B (CHB) are at a risk of developing cirrhosis and hepatocellular carcinoma (HCC) [1]. Previous studies have shown that various pre-S1/S2 mutations, including deletions and start codon mutations, accumulate in patients at later stages of chronic HBV infection and during fulminant hepatitis [2–5]. The role and effects of pre-S/S deletion mutants on mitochondrial dysfunction and HCC development have been studied longitudinally in humans, in vitro HCC cell lines, and FRG mouse models in our previous report [5]. CHB patients with pre-S/S deletion mutants are at a higher risk of HCC development in a longitudinal follow-up; however, antiviral therapy could significantly reduce HCC risk compared to those without antiviral therapy [5]. In our previous horizontal case–control study [4], six single nucleotide variations (SNVs) in genotype B HBV and twenty-one SNVs in genotype C patients in the pre-S/S region were associated with HCC. However, the roles of SNVs mutations in pre-S/S regions associated with HCC development have rarely been reported in longitudinal studies, and the efficacy of antiviral therapy in these patients remains unclear.

Current HBV treatment guidelines for non-cirrhotic patients, especially the American [6] and Asian-Pacific treatment guidelines for CHB [7], focus on patients with high viral loads and high alanine aminotransferase (ALT) levels, whereas the latest European [8] and World Health Organization (WHO) [9] guidelines recommend the expansion of antiviral therapy under certain conditions (Supplementary Table 1). However, normal or minimally elevated ALT levels in CHB patients with high viral loads do not necessarily indicate the absence of necroinflammatory activity in the liver [10, 11] and these patients remain at risk of cirrhosis or HCC [12, 13]. A recent interim analysis of the ATTENTION trial [14] suggested that early treatment with tenofovir alafenamide (TAF) reduces the risk of liver-related serious adverse events, including HCC, compared with observation in non-cirrhotic CHB patients with moderate or high viremia but normal or mildly elevated ALT concentrations. Furthermore, our earlier study [15] disclosed that CHB patients in the gray zone who did not meet with current Asian-Pacific treatment guidelines (serum HBV DNA levels < 2000 IU/mL or ALT levels < 80 U/L) but had high risk factors for HCC development (older age, male gender, HCC family history and/or serum HBV DNA levels ≥ 2000 IU/mL) were still at risk of HCC development. Antiviral therapy significantly reduced HCC incidence in gray zone CHB patients who had high risk scores [15]. However, the molecular mechanisms of hepatocarcinogenesis in gray zones CHB patients with either low HBV DNA or ALT levels are obscure.

In this longitudinal case–control study, we aimed to investigate the role of SNVs in the pre-S/S region for HCC development, the effects of HBV on mitochondrial dysfunction, and the role of antiviral therapy in patients with HCC-associated SNVs in the gray zone of the current Asian-Pacific guidelines. These findings suggest that existing guidelines could be expanded to allow for early antiviral therapy in these patients.

## Materials and methods

The detailed materials and methods are described in the Supporting Information.

### Patient enrollment

We retrospectively reviewed 104 consecutive non-cirrhotic patients with CHB in the gray zone, who underwent serum HBV DNA sequencing. These patients were mainly followed up by Prof. Jaw-Ching Wu at Taipei Veterans General Hospital and Prof. Ting-Tsung Chang at National Cheng Kung University Hospital between 1994 and 2014, who had been HCC-naïve for at least six months before enrollment (Supplementary Fig. 1). In our previous study [4], the percentage of SNVs clones that accounted for ≥20% of the total viral populations was significantly associated with HCC using next-generation sequencing, sequencing of multiple clones, or direct sequencing (detection limit of 20%). In this longitudinal study, we also used HCC-associated SNVs, accounting for 20% of viral populations at baseline of enrollment, as a cutoff value for the prediction of HCC development during the follow-up period. The study was approved by the Institutional Review Board of the participating institutes (2021-02-013BC).

### Generation of HBV SNVs DNA plasmids

HBV whole-genome clones, TW1138 and D347, were obtained via PCR amplification from patient samples, and a 1.34mer replication-competent HBV DNA plasmid was generated and cloned into the pcDNA3.1 vector. Site-directed mutagenesis was used to create SNV constructs, which were confirmed using Sanger sequencing (Supplementary Fig. 2 and Supplementary Fig. 3).

### Statistical analysis

Statistical analyses of the patient data were performed using SAS (version 9.4) and SPSS (version 20.0; SPSS, Chicago, IL).

## Results

### Baseline characteristics of the study population

The baseline characteristics of the 104 patients in the gray zone of the current treatment guidelines are shown in Table 1. The median age was 47.9 years (IQR 39.0–58.5), with a predominance of male patients (62.5%). Nearly one-third of the patients tested positive for HBeAg (36.5%) and had a family history of HCC (30.3%). Forty-three patients (41.3%) with fatty liver disease and other comorbidities are listed in Table 1. The median log HBV DNA levels were 5.3 (IQR 3.9–7.1) IU/ml and ALT was 44 (IQR 32–64) IU/ml. Among all patients, eighty-two (78.8%) received antiviral therapy during a median follow-up period of 10.4 years. The 3-, 5-, and 10-year cumulative incidence rates of HCC are 7, 9, and 14%, respectively (Supplementary Fig. 4A). Besides, patients with HCC-associated SNVs had higher cumulative incidence rates of HCC (log-rank *p* = 0.037; Supplementary Fig. 4B). Notably, antiviral therapy significantly decreased the occurrence of HCC including those with HCC-associated SNVs (Supplementary Fig. 4C and 4D).

**Table 1:** Baseline characteristics of patients in the gray zone of current treatment guidelines

Variables	Overall (n = 104)		
Demographics			
Age (year-old)	47.9 (IQR 39.0–58.5)		
Male gender, n (%)	65 (62.5)		
Genotype B/C, n (%)	75/29 (72.1/27.9)		
HBeAg positive*, n (%)	35/96 (36.5)		
Family history of HCC*, n (%)	27/89 (30.3)		
Hypertension, n (%)	19 (18.3)		
Diabetes mellitus, n (%)	6 (5.8)		
Alcohol use, n (%)	4 (3.8)		
Fatty liver, n (%)	43 (41.3)		
Immunosuppressants use^#^, n (%)	2 (1.9)		
FIB-4	1.10 (IQR 0.70–1.80)		
APRI	0.36 (IQR 0.23–0.53)		
Anti-viral therapy	82 (78.8)		
Biochemical factor			
Log HBV DNA (IU/mL)	5.3 (IQR 3.9–7.1)		
AST (IU/mL)	32 (IQR 24–45)		
ALT (IU/mL)	44 (IQR 32–64)		
Total-bilirubin (mg/dL)	0.7 (IQR 0.5–0.9)		
Platelet (10^3^/μL)	214 (IQR 172–253)		
Albumin (g/dL)	4.5 (IQR 4.3–4.7)		
AFP (ng/mL)	4.8 (IQR 3.2–7.3)		

*AFP* alpha-fetoprotein, *ALT* alanine aminotransferase, *APRI* AST to platelet ratio index, *AST* aspartate aminotransferase, *FIB-4* fibrosis-4, *HBV* hepatitis B virus, *HCC* hepatocellular carcinoma, *qHBsAg* quantitative hepatitis B surface antigen

*Missing data at the time of enrollment for this parameter

^#^One patient used cyclosporine for skin disease while and the other used steroids for rheumatologic disease

### Analysis of HCC-associated SNVs of Pre-S/S region in CHB patients of the gray zone in longitudinal follow-up

Considering that HCC-associated SNVs discovered in the horizontal study may not necessarily have a causal effect on HCC and the role of SNVs may be confounded by hepatitis flares in CHB patients who meet treatment guidelines. In addition, patients in the gray zone without high HBV DNA and ALT levels still have a risk of HCC development. Therefore, we investigated the association between HCC and candidate SNVs in the pre-S/S region from our previous horizontal case–control study [4] including six in genotype B and 21 HCC-associated SNVs in genotype C for those patients in the gray zone. Overall, patients with genotype B had lower HBeAg positivity (28.2 vs. 60.0%, *p* = 0.004), a lower Fibrosis index based on the 4 factors (age, platelet counts, ALT, AST; FIB-4) score (1.00 vs. 1.36, *p* = 0.049), a lower AST to Platelet Ratio Index (APRI) score (0.33 vs. 0.50, *p* = 0.005), less antiviral therapy use during follow-up (73.3 vs. 96.6%, *p* = 0.008), lower HBV DNA levels (4.8 vs. 7.1 log IU/mL, *p* = 0.001), lower AST (29 vs. 41 U/L, *p* = 0.001), lower ALT levels (44 vs. 57 U/L, *p* = 0.043), and higher albumin levels (4.5 vs. 4.3 g/dL, *p* = 0.018) compared with genotype C patients (Supplementary Table 2). HCC occurred significantly often in gray zone patients with T53C SNV, A273G SNV, and A529G SNV of genotype B HBV-patients (Supplementary Table 3) while frequently with T53C SNV, G633A SNV, and A3120G SNV of genotype C HBV-infected patients (Supplementary Table 4).

To further determine the risk factors for HCC in the gray zone, the analysis workflow consisted of two stages: (1) initial variable selection using LASSO regression to identify independent predictors of HCC risk and (2) construction of a multivariable time-dependent Cox model incorporating the selected covariates, with antiviral therapy use as a time-varying exposure. Univariate analyses revealed that older age, absence of antiviral therapy, higher AFP levels, presence of candidate HCC-associated SNVs, and pre-S/S deletion were significantly associated with an increased risk of HCC. Multivariate analysis revealed that older age (hazard ratio [HR] = 1.105, 95%CI: 1.041–1.179; *p* = 0.001), no antiviral therapy (HR = 0.195, 95%CI: 0.060–0.637; *p* = 0.007), and occurrence of candidate HCC-associated SNVs (HR = 1.863, 95%CI: 1.208–6.871; *p* = 0.028) were factors associated with HCC (Table 2).

**Table 2:** Cox’s proportional hazards model for predictors of HCC occurrence in the patients of gray zone

Variables	Univariate			Multivariate		
	HR	95%CI	*p* value	HR	95%CI	*p* value
Age (per year increase)	1.122	1.057–1.192	<0.001	1.105	1.041–1.179	0.001
Male (vs. female)	1.470	0.460–4.693	0.516			
Genotype C (vs. B)	1.327	0.418–4.208	0.631			
HBeAg positive (vs. negative)	8.256	0.961–64.25	0.074			
Family history of HCC (vs. no)	1.085	0.423–2.649	0.195			
Hypertension (vs. no)	0.827	0.185–3.704	0.804			
Diabetes mellitus (vs. no)	3.423	0.762–15.38	0.108			
Alcohol use (vs. no)	5.489	0.722–24.65	0.126			
Fatty liver (vs. no)	0.504	0.160–1.587	0.242			
Immunosuppressants use (vs. no)	0.048	0.00-infinity	0.719			
Time-varying anti-viral therapy (vs. no)	0.161	0.050–0.570	0.005	0.195	0.060–0.637	0.007
HBV DNA ≥ 2000 (vs. <2000 IU/mL)	1.906	0.852–3.252	0.179			
ALT ≥ 2 × ULN (vs. <2 × ULN)	22.81	0.00–infinity	0.478			
AST (U/L)	1.005	0.997–1.013	0.203			
Platelet (×10^3^/μL)	0.988	0.975–1.001	0.067			
Total bilirubin (mg/dL)	1.340	0.722–2.488	0.353			
Albumin (g/dL)	0.164	0.024–1.124	0.066			
AFP (ng/ml)	1.023	1.008–1.038	0.002			
Candidate HCC-associated SNVs (vs. no)	1.755	1.482–6.394	0.024	1.863	1.208–6.871	0.028
Pre-S/S deletion (vs. no)	2.198	1.090–6.117	0.031			

*AFP* Alpha-fetoprotein, *ALT* alanine aminotransferase, *APRI* AST to platelet ratio index, *AST* aspartate aminotransferase, *FIB-4* fibrosis-4, *HBV* hepatitis B virus, *HCC* hepatocellular carcinoma, *SNV* single-nucleotide variants, *ULN* upper limit of normal

**Fig. 1:**
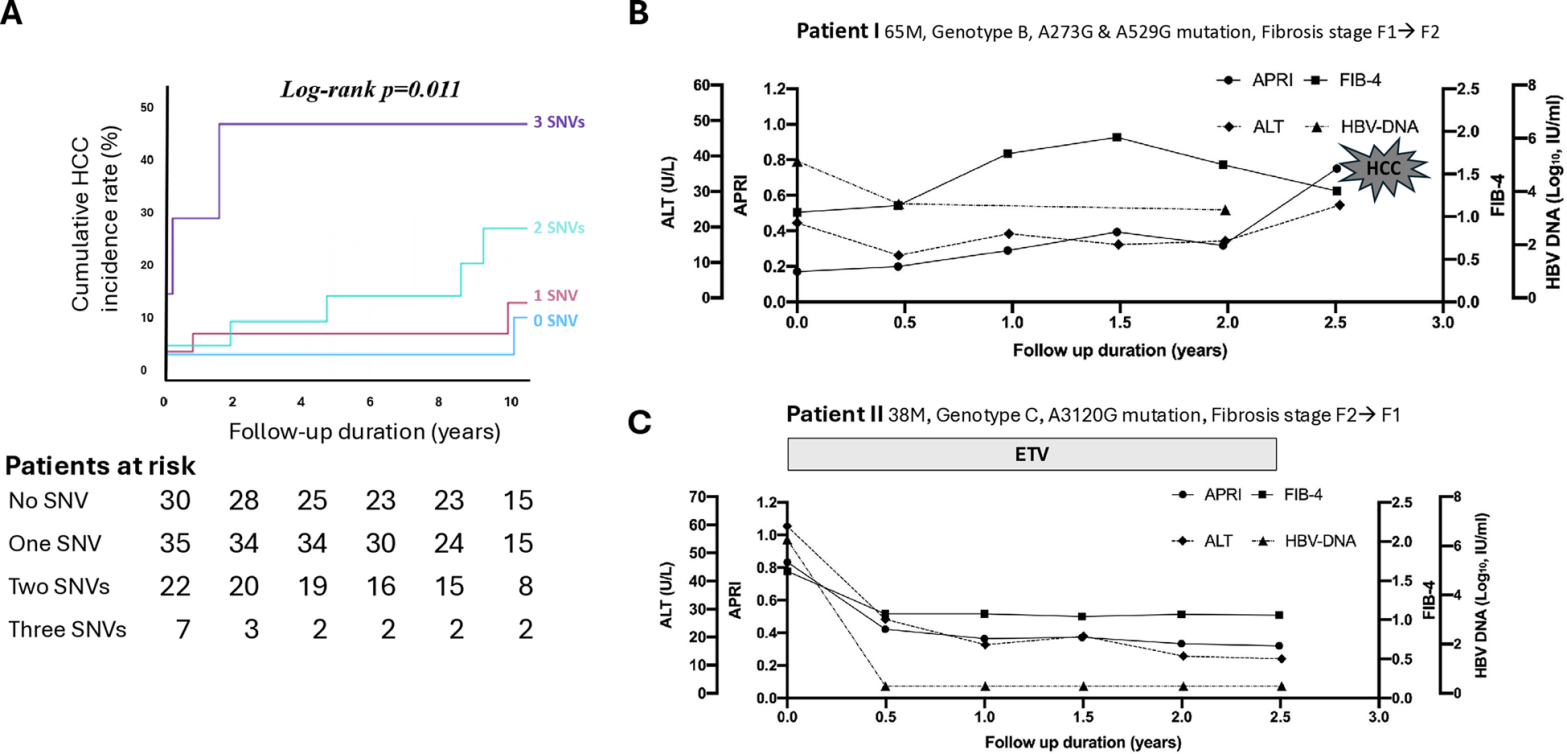
Association between HCC-associated SNVs and HCC occurrence. **A** Patients carrying all three HCC-associated SNVs had the highest HCC incidence rate in the entire cohort (log-rank *p* = 0.011). **B**, **C** Clinical course and outcomes of representative cases with HCC-associated SNVs, with and without antiviral therapy

After defining the three candidate HCC-associated SNVs in patients with genotype B and C, we investigated whether synergistic effects occurred when these SNVs coexisted. The cumulative HCC incidence was higher when patients co-existed with all three SNVs (3-, 5-, and 10-year of 3 vs. 2 vs. 1 vs. 0 SNVs: 48% vs. 9% vs. 7% vs. 3%, 48% vs. 14% vs. 7% vs. 3%, and 48% vs. 27% vs. 12% vs. 3%, respectively; log-rank *p* = 0.011; Fig. 1A). The representative clinical courses and outcomes of patients with candidate HCC-associated SNVs are shown in Fig. 1B, C Patient 1 showed serum genotype B HBV with the A273G mutation, accompanied by a high viral load (5.9 log IU/ml at baseline) and normal ALT levels that did not meet the reimbursement criteria for antiviral treatment. The risk score was 9 based on our previous high-risk scores (≥8) for predicting HCC in the gray zone [15]. Slow progression of fibrosis scores was noted, and the patient developed HCC in the absence of cirrhosis 2.5 years later after presentation. In contrast, patient 2 showed genotype C HBV with the A3120G mutation, accompanied by a high viral load (7.2 log IU/mL at baseline) and mildly elevated ALT levels (58 IU/mL at baseline) with a risk score of 9 [15]. The patient agreed to receive self-paid antiviral therapy due to concerns of progression to cirrhosis or HCC with a family history of HCC. The fibrosis scores slowly declined to mild fibrosis F1, and no HCC occurred during the 3-year follow-up period. Of note, antiviral therapy significantly decreased the occurrence of HCC in patients with and without the co-existence of any SNVs. There was a trend of reduction in HCC in treated patients, but it did not reach statistical significance in those co-existing with all three HCC-associated SNVs because of the limited number of cases (Supplementary Fig. 5). Among patients with HCC-associated SNVs, those treated with antiviral therapy were more likely to have higher AST and ALT levels. There was a trend toward higher APRI scores in patients receiving antiviral therapy (Supplementary Table 5).

Among the 15 patients who developed HCC during the follow-up period, three patients (20.0%) based on the FIB-4 score and five patients (33.3%) based on the APRI score demonstrated a fibrosis stage < F2 at enrollment; the remaining patients had fibrosis stage ≥ F2 based on the APRI score or FIB-4, and thus were supposed to meet the WHO criteria. All these patients were outside the current APASL HBV treatment guidelines, 9 (60%) met with the AASLD guidelines with twice upper limit of normal ALT levels (35 U/L for men and 25 U/L for women compared to 40 U/L in APASL and EASL guidelines) which also include the risk factor of HCC family history, 13 patients (87%) met the latest EASL HBV treatment guidelines. Moreover, all patients met the WHO treatment guidelines and had high-risk scores (≥8) in our previous report [15]. Eight patients (53.3%) received self-paid antiviral therapy after enrollment, and most (87.5%) had fibrosis stage ≥ F2 when antiviral therapy was initiated (Supplementary Table 6).

### The expression of HCC-associated SNVs disturbed mitochondrial dynamics and morphology

HCC-associated SNVs disrupt mitochondrial dynamics and morphology, which are critical for cellular health. Huh7 cells transfected with wild-type HBV, preS2ΔMT, or HCC-associated SNV plasmids showed altered mitochondrial function via MitoTracker Red labeling and live-cell imaging (Supplementary Videos 1–9). Vector-transfected cells had elongated, dynamic mitochondria, whereas HBV clones (D347, TW1138) and SNVs (A293G, G633A, T53C, T216C, A273G) displayed fragmented, spherical mitochondria with diminished motility. Immunofluorescence co-labeling with MitoTracker Red and HBsAg further confirmed the fragmented morphology in HBV-expressing cells across both genotypes B and C (Supplementary Fig. 6A and 6B).

### The expression of HCC-associated SNVs reduced mitochondrial membrane potential and increased ROS level

Mitochondria are the main source of ROS production in cells [16]. In pathological conditions associated with mitochondrial respiratory chain dysfunction, decreased mitochondrial membrane potential, decreased respiratory chain activity, and increased ROS production have been observed [17]. Therefore, we analyzed the changes in mitochondrial membrane potential and ROS production caused by SNVs expression in Huh7 cells.

Cells expressing WT HBV from both genotypes B (D347) and C (TW1138) exhibited significantly reduced mitochondrial membrane potential compared to the no-virus vector control. Cells expressing HCC-associated SNVs (A293G, A3120G, and T53C in genotype C; T53C, A273G, and A529G in genotype B) showed an even greater reduction in mitochondrial membrane potential than those expressing WT HBV (Supplementary Fig. 7A). FCCP, an agent that uncouples oxidative phosphorylation in the mitochondria, was used as a positive control to indicate complete loss of mitochondrial membrane potential. Similarly, ROS levels were significantly higher in cells expressing WT HBV compared to the no-virus vector control. Cells with HCC-associated SNVs showed even higher ROS production than cells with WT HBV (Supplementary Fig. 7B). Antimycin-A, an inhibitor of complex III and a known ROS generator, served as a positive control.

### The effect of HCC-associated SNVs on cell metabolism

**Fig. 2:**
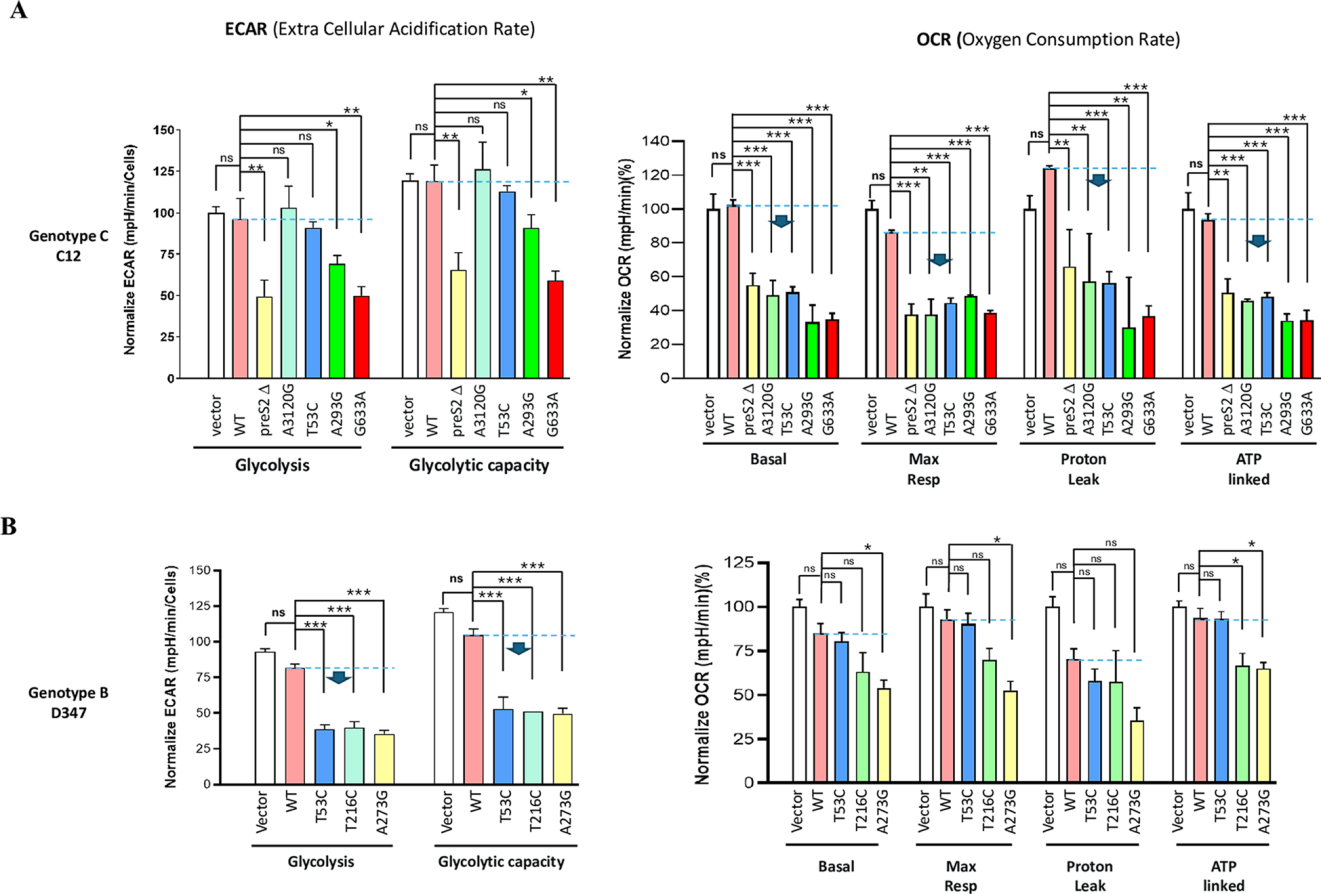
Effect of HBV SNVs on cell metabolism. Huh7 cells were transfected with HBV expression plasmids, including WT, preS2ΔMT, and SNVs of D347 (genotype B) and C12 (genotype C), for 48 h. OCR and ECAR were measured using the Seahorse XF96 instrument with Glycolysis Stress Test and Mito Stress Test, respectively. **A** Genotype C SNVs had a significant impact on OCR and a minor effect on ECAR. **B** Genotype B SNVs show a significant impact on ECAR and a minor effect on OCR. One-way ANOVA was performed using GraphPad Prism (*p < 0.05; **p < 0.01; ***p < 0.001) (*N* = 3–4)

Mitochondria are central to cellular metabolism. To assess the impact of HBV and associated SNVs on cell metabolism, we used a Seahorse extracellular flux analyzer to directly measure the oxygen consumption rate (OCR) and extracellular acidification rate (ECAR). In cells expressing HBV clone C12 (genotype C, WT-C12), the effect on ECAR or OCR was not statistically significant compared to virus-free vector controls (Fig. 2A). However, when cells expressed C12-derivated SNVs such as A3120G, T53C, A293G, and G633A, the OCR, indicative of mitochondrial respiration, was significantly inhibited by more than 50% (Fig. 2A, OCR panel). Key mitochondrial respiration parameters, including basal, ATP production-related, maximal, and proton leakage-related OCR, were significantly reduced. In contrast, the SNVs of genotype C seemed to have a much lesser effect on ECAR (representing glycolytic efficiency) (Fig. 2A, ECAR panel). C12-A3120G and C12-T53C showed no statistically significant difference compared to C12-WT. C12-A293G slightly reduced ECAR by approximately 30% in the basal glycolysis rate and by 10% in the glycolytic capacity. C12-G633A reduced ECAR the most, with approximately 50% basic glycolysis and 40% glycolysis capacity (Fig. 2A). Another HBV clone of genotype C, TW1138, showed similar Seahorse metabolic flux assay results as C12 (see Supplementary Fig. 8).

In cells expressing the HBV clone D347 (genotype B), D347-WT showed no statistically significant effect on ECAR or OCR compared to the virus-free controls (Fig. 2B). However, cells expressing SNV clones, such as D347-T53C, T216C, and A273G, exhibited a significant reduction in ECAR by 65% in basal glycolytic capacity and by 50% in glycolytic capacity (Fig. 2B, ECAR panel). Unlike C12, the D347-derived SNVs did not significantly affect the OCR (Fig. 2B, OCR panel). D347-T53C and T216C were not significantly different from D347-WT. D347-A273G reduced OCR the most, with a statistical significance of a p value of approximately 0.05.

**Fig. 3:**
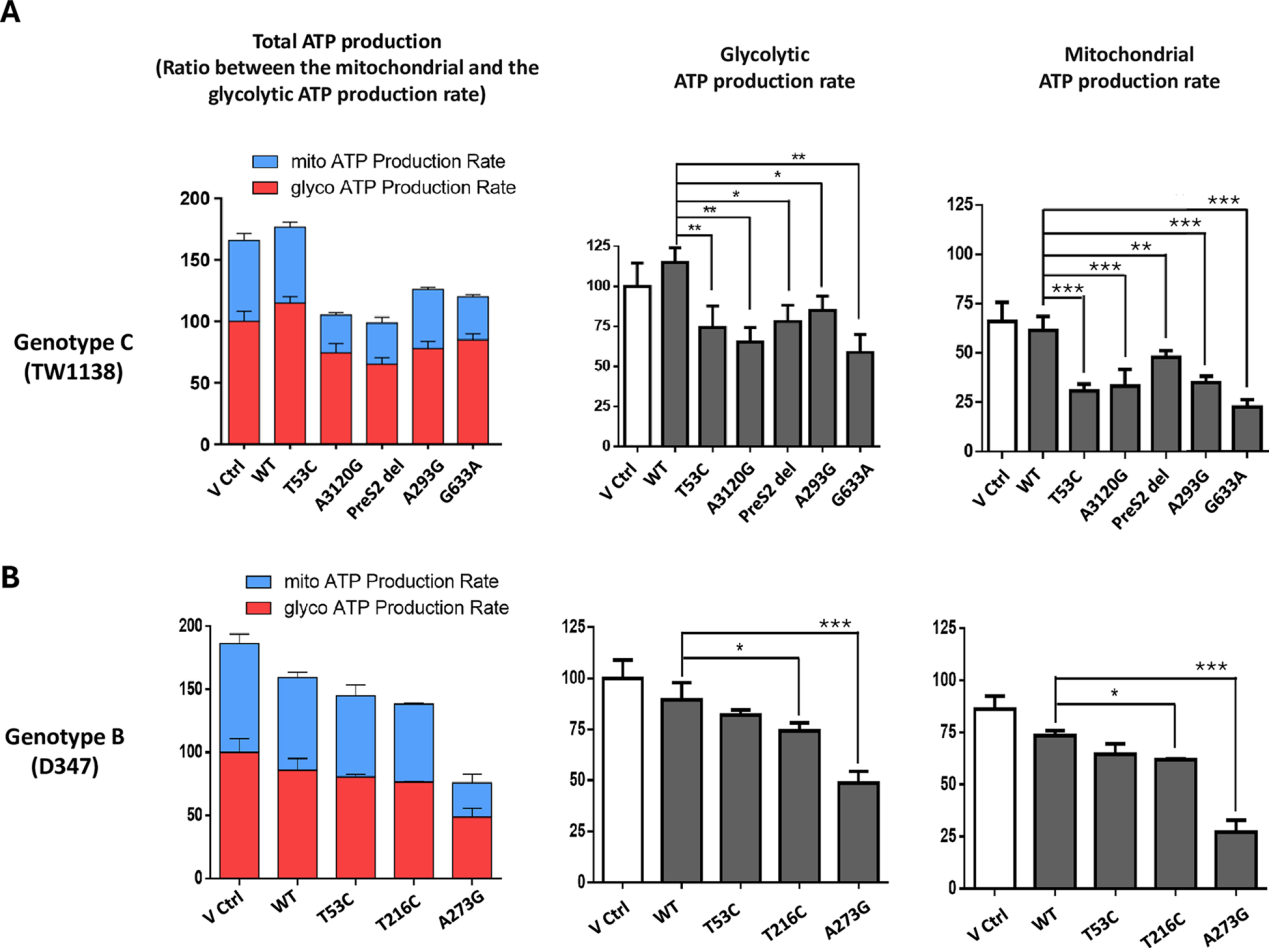
The expression of HBV SNVs decreased ATP production. WT or SNV HBV expression plasmids for genotype C (clone TW1138) and B (clone D347) were transfected into Huh7 cells for 48 h. Cellular ATP production was measured using the Seahorse XF real-time ATP rate analysis. **A** Genotype C SNVs had a more substantial impact on mitochondrial ATP production and a minor effect on glycolytic ATP production. **B** Genotype B SNVs affected mitochondrial and glycolytic ATP production equally. Statistical analysis was performed using one-way ANOVA in GraphPad Prism (*p < 0.05; **p < 0.01; ***p < 0.001) (*N* = 4)

We further analyzed the proportion of ATP produced from cytosolic glycolysis and mitochondrial oxidative phosphorylation using a real-time ATP production rate assay with the Seahorse extracellular flux analyzer. For genotype C, the overall ATP production rate was reduced in cells expressing the WT HBV and SNVs (Fig. 3A, left panel). This reduction was primarily due to a significant decrease in ATP derived from mitochondrial oxidative phosphorylation (Fig. 3A, right panel), with only minor changes in ATP derived from glycolysis (Fig. 3A, middle panel). In contrast, for genotype B (Fig. 3B), the reduction in the total ATP production rate in cells expressing WT HBV and SNVs was smaller, with similar reduction rates in ATP production from both glycolysis and oxidative phosphorylation.

Overall, these results suggest that SNVs of genotype C may have a more substantial impact on mitochondrial function, potentially leading to greater reliance on glycolysis for cellular energy, implying that the Warburg Effect occurs in these cells. In contrast, SNVs of genotype B may maintain better mitochondrial function, indicating relatively sufficient cellular energy (Figs. 2 and 3).

### The expression of HCC-associated SNVs disrupt cellular calcium homeostasis

Several studies, including our previous study, have shown that HBV can elevate cytosolic Ca^2+^ levels [5, 18]. In this study, we further examined how WT HBV- and HCC-associated SNVs alter the kinetic calcium response to ATP-induced cytosolic calcium changes in Huh7 cells. Using Fura-2 calcium indicators and laser-scanning confocal microscopy, we pre-loaded the cells with Fura-2 in calcium-containing buffer. Cytosolic calcium signals were triggered by adding 100 µM ATP, and changes in Ca^2+^ levels were monitored by alternately exciting Fura-2 at 340 and 380 nm. The ratio of emissions at these wavelengths is directly related to the amount of intracellular calcium.

**Fig. 4:**
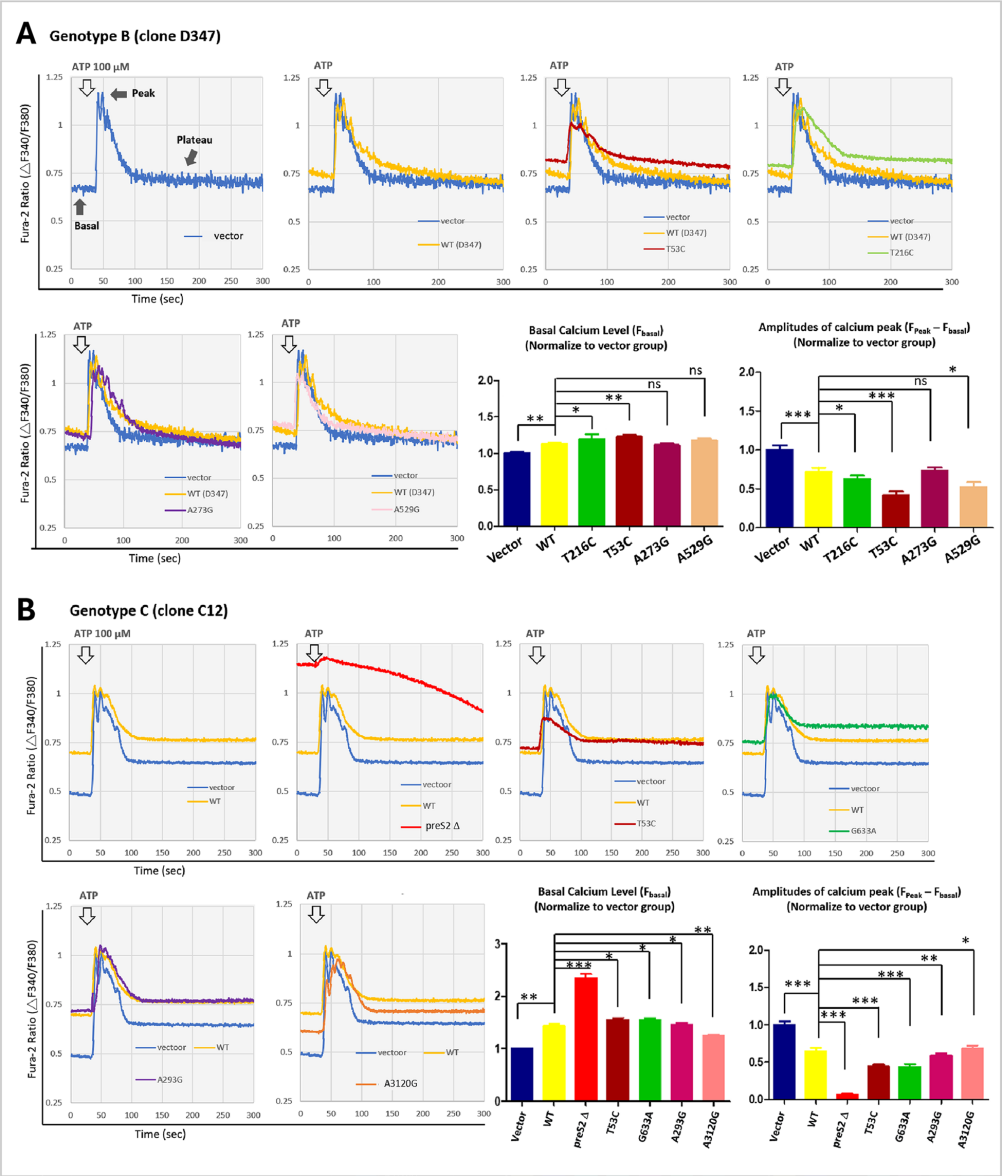
WT HBV and HCC-Associated SNVs Affect the Kinetic Calcium Response to ATP-Induced Cytosolic Calcium in Huh7 Cells. Huh7 cells were transfected with WT HBV and HCC-associated SNVs for 48 h, and the kinetic calcium response to ATP-induced cytosolic calcium changes was measured using Fura-2 calcium indicators. **A** Genotype B (clone D347) and **B** Genotype C (clone C12) plasmids, along with their respective SNVs, were used for transfection. The *open arrows* indicate the time required for ATP addition. The time course of the changes in the Fura-2 fluorescence ratio in response to 100 μM ATP was monitored at a 340/380 nm ratio. In the virus-free vector control, during ATP stimulation, *solid arrows* indicate three phases of calcium level changes: basal, peak, and plateau. The bar chart summarizes the effects of HBV variants on the basal calcium levels and amplitudes of the calcium peak (difference in calcium levels between peak and basal), calculated as (F_peak − F_basal) of SNVs/(F_peak − F_basal) of the vector

As shown in Fig. 4, mock-transfected Huh7 cells exhibited a typical IP3-linked Ca^2+^ response, with a rapid peak in Ca^2+^ signaling immediately after ATP addition, followed by a gradual recovery phase resulting in an enhanced cytosolic Ca^2+^ level, termed the plateau (see Fig. 4, “vector” panel). Compared to mock-transfected controls, expression of D347 WT or SNVs (T216C, T53C, and A273G) showed altered calcium kinetics, characterized by higher basal levels, reduced peak amplitude, and an elevated plateau, indicative of disrupted cellular calcium-buffering capacity (Fig. 4A). Conversely, cells expression of TW1138 WT and SNVs (T53C, A293G, G633A, and A3120G) exhibited a more pronounced disruption in calcium kinetics, with markedly elevated basal levels, diminished peak amplitude, and an elevated plateau (Fig. 4B). These findings suggest that both WT HBV and SNVs can significantly affect cellular calcium homeostasis, with genotype C exerting a greater effect than genotype B, resulting in impaired calcium buffering efficacy.

## Discussion

In this longitudinal case–control study, we examined HCC development in gray-zone CHB patients with or without pre-S/S SNVs and the impact of antiviral therapy. Several HCC-specific SNVs, particularly T53C, were associated with HCC in both HBV genotypes B and C. Patients with moderate or high viral load benefited from early antiviral therapy regardless of ALT levels which is consistent with the ATTENTION trial and our previous work [14, 15]. Notably, some CHB patients in the gray zone who later developed HCC would have been eligible for treatment under the latest WHO guidelines and our previously defined high HCC-risk score (≥8) [9, 15, 19]. However, they were outside strict APASL criteria, 40% outside AASLD, and 13% outside the latest EASL guidelines [6–8]. This deferred treatment likely contributed to disease progression and eventual HCC.

Our previous report showed that initiating therapy before age 40 and prior to cirrhosis provided the greatest protection [20]. Among patients in the gray zone, HCC-associated SNVs promoted fibrosis in a dose-dependent manner and could drive HCC development even without cirrhosis if untreated. In contrast, timely antiviral therapy stabilized or reduced fibrosis and prevented HCC during follow-up (Fig. 1), even in patients carrying HCC-associated SNVs (Supplementary Fig. 4). Nonetheless, some patients still progressed, likely due to late treatment initiation in older individuals with fibrosis ≥ F2 and additional risk factors such as age ≥ 50, male sex, or a family history of HCC [15].

The pre-S regions contain multiple T- and B-cell epitopes, highlighting their role in immune recognition; mutations in these regions may enable viral escape from immune surveillance, whereas wild-type HBV is gradually cleared by host immunity [21, 22]. In our study, the nt53 mutation in the pre-S2 region (F141L) altered both B- and T-cell epitopes and was associated with HCC development, consistent with findings by Mun et al. [23]. In this study, A3120G (A91A) and T53C (F141L) in both genotypes B and C were significant S-region mutations associated with HCC in gray-zone CHB patients. Additional significant mutations included A273G (N214S) and A529G (T299T) in genotype B, and G633A (R334K) in genotype C, highlighting the cumulative impact of pre-S/S variants on disease progression.

In addition, we elucidated genotype-specific differences in HCC development between HBV genotypes B and C. Metabolic analyses showed that mitochondrial and glycolytic ATP declined markedly in genotype C but only mildly in genotype B, potentially explaining the differential HCC risk (Supplementary Fig. 7). Clinically, genotype B HBV-infected patients in the gray zone had lower HBeAg positivity, serum HBV DNA levels, and fibrosis scores than genotype C patients, consistent with reports linking genotype C to higher viral load and increased HCC risk without antiviral therapy [24, 25]. Future studies should investigate genotype-specific mtDNA damage and whether early antiviral therapy at lower fibrosis stages can reverse mitochondrial dysfunction and reduce HCC incidence in gray-zone CHB patients.

Pre-S/S mutations are common in chronic HBV infection and strongly linked to HCC [2, 5, 21, 22, 26–28]. These variants often impair HBsAg secretion, leading to intracellular retention, ground-glass hepatocytes, and minimal ALT elevation [5, 29]. Accumulated HBsAg induces ER stress and activates the unfolded protein response (UPR), initially promoting cell survival by engaging adaptive pathways [30–33]. If unresolved, the PERK–ATF4–CHOP and IRE1–JNK branches induce BH3-only proteins, activate BAX/BAK, cause mitochondrial outer membrane permeabilization, and ultimately lead to caspase-mediated apoptosis [5, 34–39]. These stress responses are exacerbated by Ca^2^⁺ flux at ER–mitochondria contact sites, mitochondrial ROS production, and mtDNA damage [40, 41]. Together, these pathways explain how HBV, especially mutants with misfolded HBsAg, can drive hepatocarcinogenesis even in patients with low HBV DNA and ALT levels.

Our findings of A3120G, T53C, and other S-region SNVs being associated with HCC are consistent with previous reports showing that pre-S/S mutations promote ER stress, oxidative DNA damage, and fibrosis [5, 23, 27, 42]. In a humanized mouse liver model with human hepatocytes, preS2-deleted HBV induced stronger ER stress and activation of the UPR, leading to enhanced apoptosis, and progressive fibrosis compared with wild-type HBV, despite lower serum HBV DNA and HBsAg secretion [5, 34, 37–39]. In HBV-transfected cell models, we observed similar mitochondrial dysfunction, with excessive ROS and decreased ATP production—especially in genotype C, which correlates with its higher clinical HCC risk. These observations are also supported by studies showing that HBV or HBx mutants increase mitochondrial oxidative stress, mtDNA damage, and metabolic reprogramming in human HCC tissues [40, 43].

Importantly, antiviral therapy alleviates HBV-induced ER stress, UPR activation, and mitochondrial dysfunction in both experimental and clinical settings. In this study, tenofovir alafenamide (TAF) protected HBV-infected hepatoma cells from ROS accumulation and mitochondrial depolarization (Supplementary Fig. 9), consistent with our prior reports showing that antivirals restore autophagy, reduce ER stress, and lower HCC incidence in mouse models [34]. These findings suggest that antiviral therapy not only suppresses viral replication but also mitigates SNV-driven ER–mitochondria dysfunction, supporting earlier intervention in high-risk gray-zone patients.

Notably, in our in vitro transfection system, HBV whole-genome DNA was introduced into Huh7 or HepG2 cells, enabling viral replication and viral particle production. While TAF does not directly inhibit HBsAg production from integrated HBV DNA, it ultimately reduces overall viral load by inhibiting viral genome replication and subsequently decreases HBsAg levels. However, HBsAg levels usually do not show a rapid decline after TDF treatment because the amounts of HBsAg derived from both episomal and integrated HBV DNA are usually more than 1000–10000 folds than those of HBV particles. In transfected cells, there is no integrated HBV DNA. HBV replication and HBsAg expression are produced from non-integrated episomal HBV DNA. Further evidence is needed to determine the amount of HBsAg expression of pre-S mutations inhibited in very short time transfection system after TAF administration.

Currently, in the absence of effective strategies to eliminate integration-derived HBsAg, antiviral therapy remains the most reliable approach to counteract virus-induced liver injury and its progression to HCC in the HBV-infected population. The protective effects observed in our cell model are consistent with the long-term clinical benefits of antiviral therapy in reducing HCC risk, which are well established [20].

Subtle mitochondrial and metabolic dysfunctions may go unnoticed and untreated under current criteria that require high HBV DNA and ALT levels. Immune evasion and secretion-defective pre-S deletion or SNV HBV mutants lead to intracellular retention of HBV and HBsAg in the ER, causing liver injury not reflected by serum ALT levels [5, 15, 34, 40, 44]. Antiviral therapy can counter hepatocarcinogenesis by suppressing HBV replication and restoring mitochondrial and metabolic function, as shown in human and in vivo studies [5, 15, 40]. These findings support expanding antiviral treatment to CHB patients outside current Asia–Pacific guidelines, and will benefit these patients from developing HCC or liver-related deaths.

This study is limited by the small number of patients in some subgroups, which reduced statistical power and produced non-significant trends in certain analyses. The hazard ratio confidence intervals for candidate HCC-associated SNVs were relatively wide, reflecting imprecision in estimation due to the limited sample size. The HCC risk score system in CHB patients of the gray zone were based on hospital cohorts from medical centers had been externally validated by the REVEAL community cohort of 3527 CHB patients in the gray zone gray zone [15]. Future studies in larger gray-zone patient cohorts from hospital and community settings are required to validate the role of HBV SNVs in HCC development.

Universal SNV screening may be challenging and impractical in routine clinical practice for gray-zone CHB patients. In addition, mechanistic insights regarding SNV-induced mitochondrial dysfunction are primarily derived from cell models. Therefore, HCC-associated SNVs should be regarded as biomarkers rather than definitive causative factors. Residual confounders—including age, sex, fibrosis stage, family history, and host or viral variations—may also influence HCC development.

Rather than advocating universal screening, our study emphasizes that gray-zone CHB patients—often carrying HCC-associated SNVs—should not be considered truly “low-risk.” Our findings suggest a potential role of SNV-driven mitochondrial dysfunction in hepatocarcinogenesis and indicate that such patients may benefit from earlier antiviral therapy.

Our in vitro system produced HBsAg concentrations (15–25 IU/mL) much lower than those of most CHB patients but close to some gray zone patients (ranged from <100 to >1000 IU/mL). It is a limitation of in-vitro assay to exactly recapitulate all CHB patients at different stages, however, it alleviates concerns of artificial overexpression.

## Conclusion

Our long-term follow-up study demonstrates that gray-zone patients remain at risk of HCC due to pre-S/S SNVs associated with mitochondrial dysfunction and metabolic changes. These mutant-related injuries progress despite low HBV DNA and ALT levels, driven by secretion-defective and immune-escape variants. Antiviral therapy reduces this risk, supporting earlier initiation, particularly in younger patients, those with a family history of HCC, or fibrosis stage ≥ F2, in line with WHO but not Asia–Pacific guidelines. Further studies are warranted to determine whether therapy should begin before F2 in high-risk gray-zone patients [15].

## Supplementary Information

Additional file 1.

Additional file 2.

Additional file 3.

Additional file 4.

Additional file 5.

Additional file 6.

Additional file 7.

Additional file 8.

Additional file 9.

Additional file 10.

Additional file 11.

Additional file 12.

Additional file 13.

Additional file 14.

Additional file 15.

Additional file 16.

Additional file 17.

Additional file 18.

Additional file 19.

## Data Availability

All data and materials are available on reasonable request to the corresponding authors.

## Abbreviations

AFP: Alpha-fetoprotein
ALT: Alanine aminotransferase
APRI: AST to platelet ratio index
AST: Aspartate aminotransferase
CHB: Chronic hepatitis B
ECAR: Extracellular acidification rates
ER: Endoplasmic reticulum
FIB-4: Fibrosis index based on the 4 factors
HBsAg: Hepatitis B surface antigen
HBV: Hepatitis B virus
HCC: Hepatocellular carcinoma
NA: Nucleos(t)ide analogue
OCR: Oxygen consumption rates
ORFs: Open reading frames
PCR: Polymerase chain reaction
qHBsAg: Quantitative hepatitis B surface antigen
ROS: Reactive oxygen species
SNVs: Single-nucleotide variants
UPR: Unfolded protein response
WT: Wild type
