# Supplementary figures and images for: The impact of single-nucleotide variants of hepatitis B virus and antiviral on liver cancer in gray zone patients
Source: J Biomed Sci. 2025 Dec 1;32:101. doi: 10.1186/s12929-025-01195-x (PMC12667080; doi:10.1186/s12929-025-01195-x)

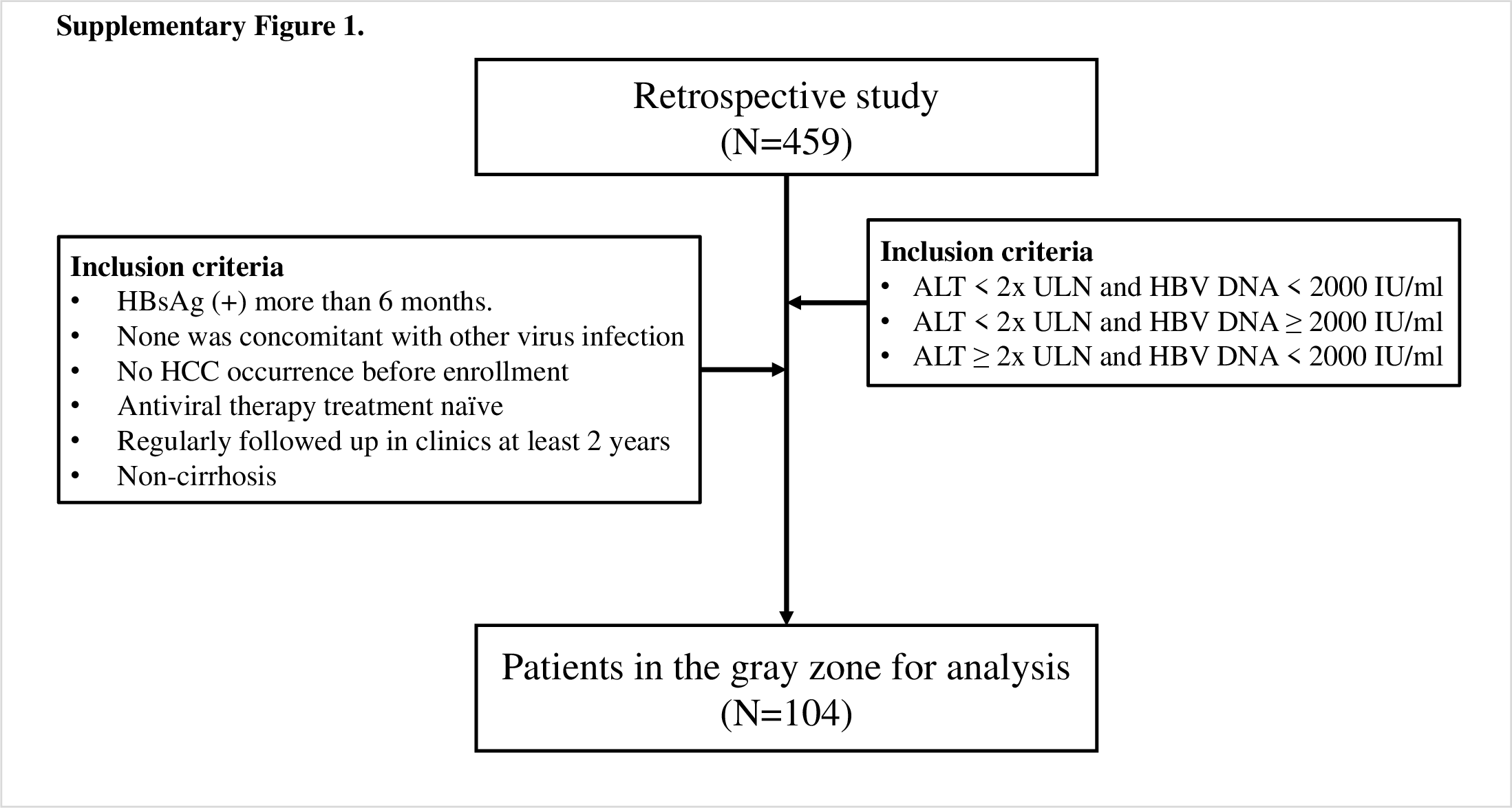

Supplement: Supplementary file 1 — Additional file 1. [file 12929_2025_1195_MOESM1_ESM.tif]

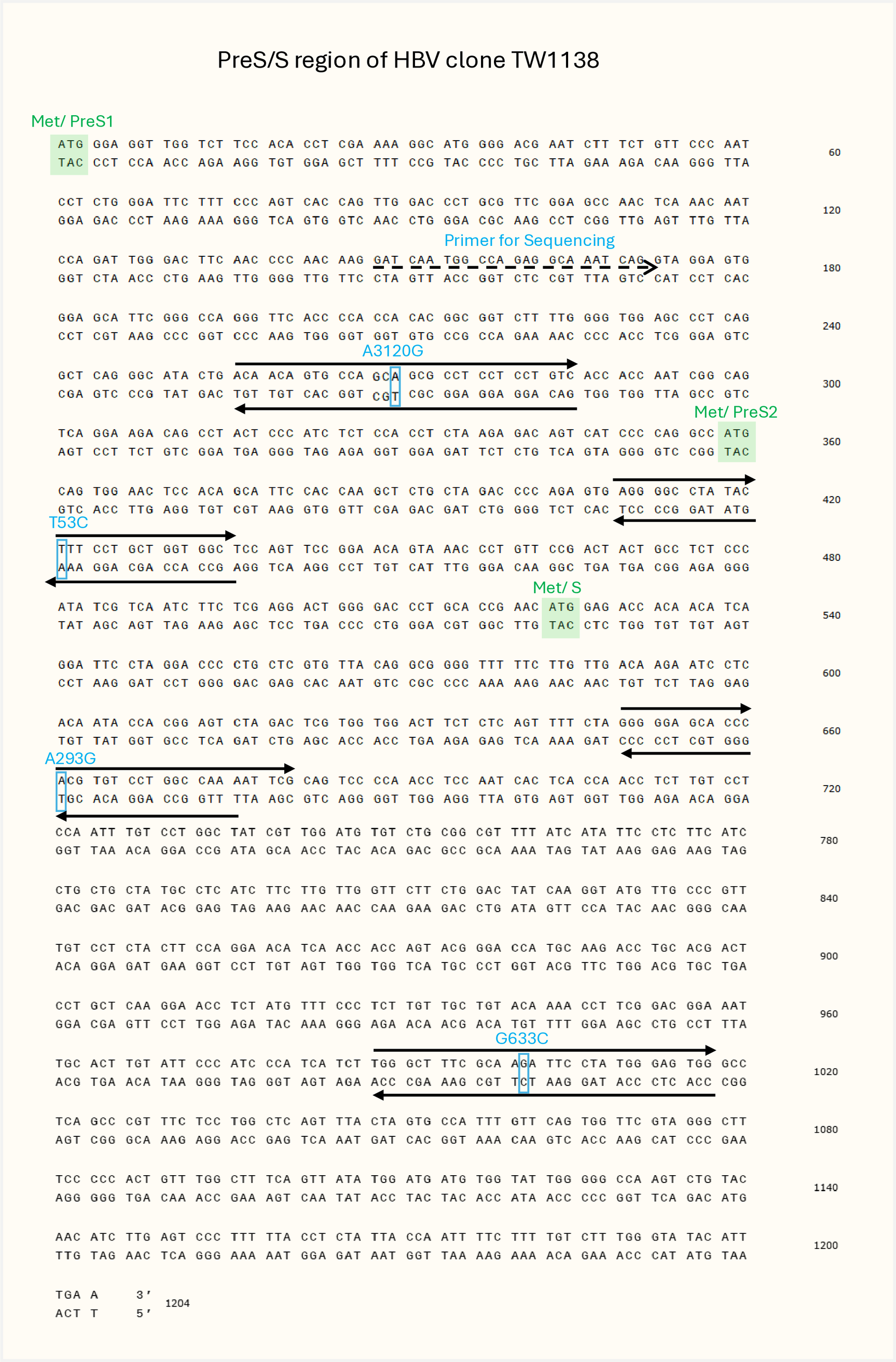

Supplement: Supplementary file 2 — Additional file 2. [file 12929_2025_1195_MOESM2_ESM.tif]

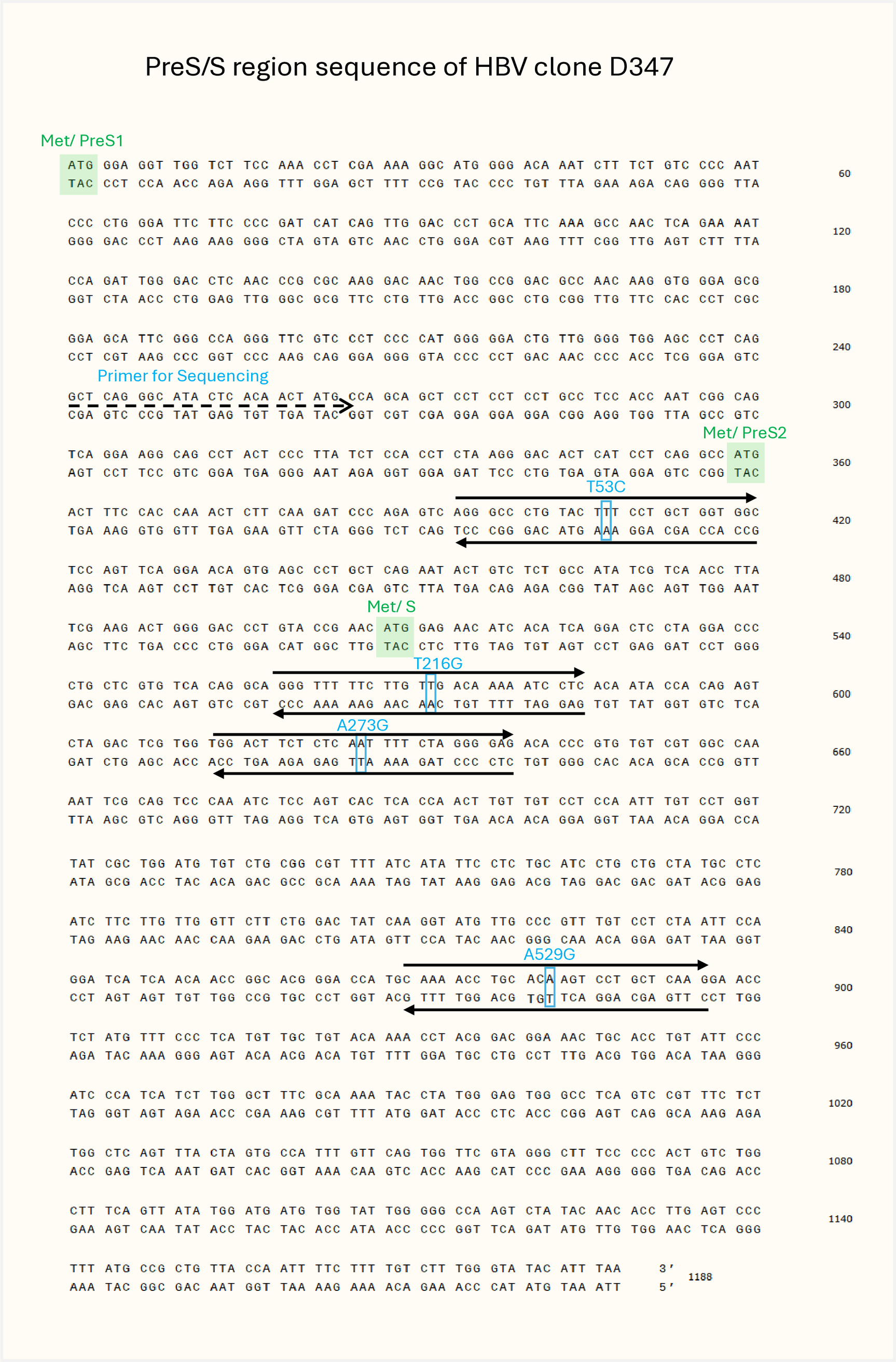

Supplement: Supplementary file 3 — Additional file 3. [file 12929_2025_1195_MOESM3_ESM.tif]

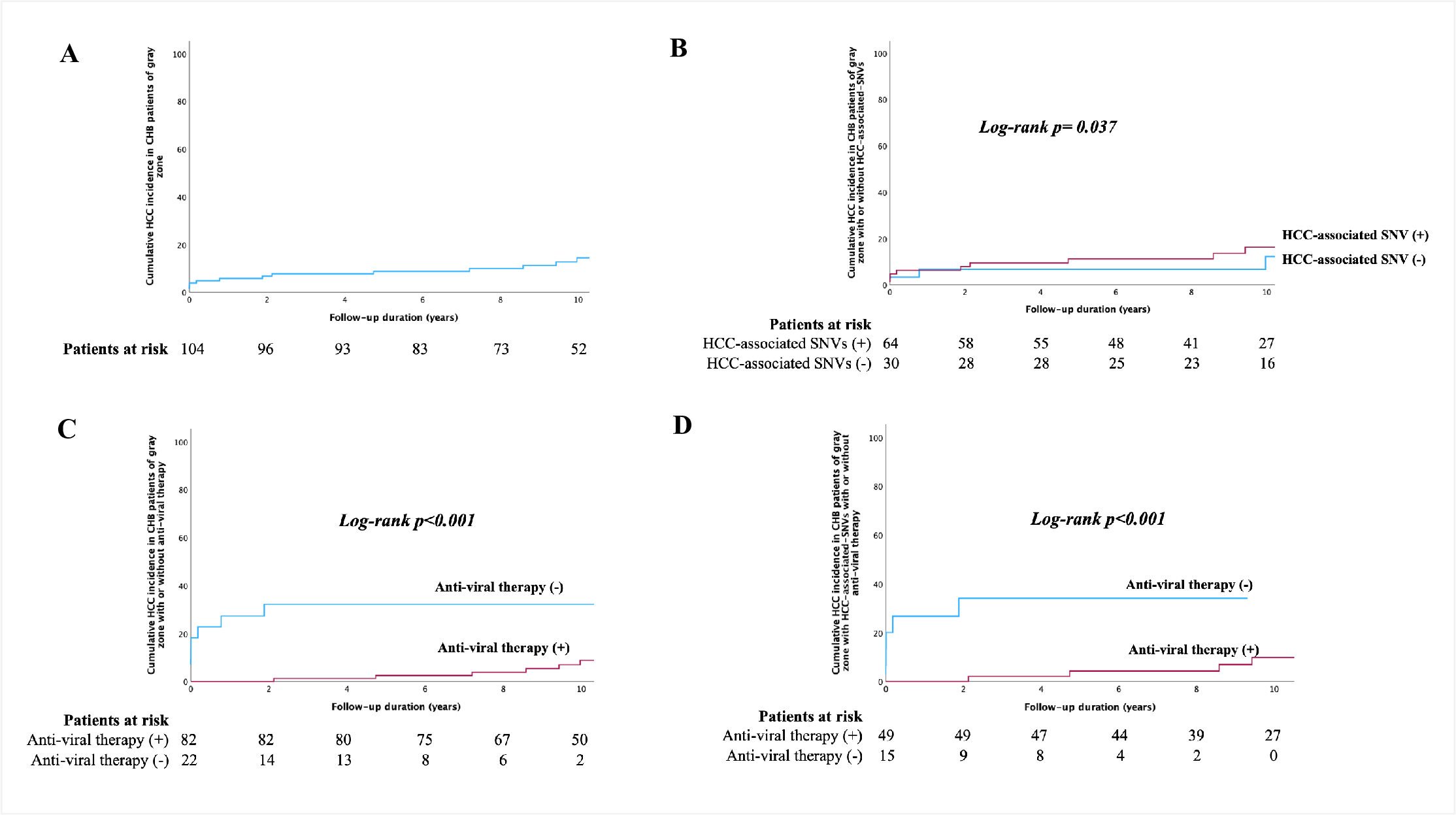

Supplement: Supplementary file 4 — Additional file 4. [file 12929_2025_1195_MOESM4_ESM.tif]

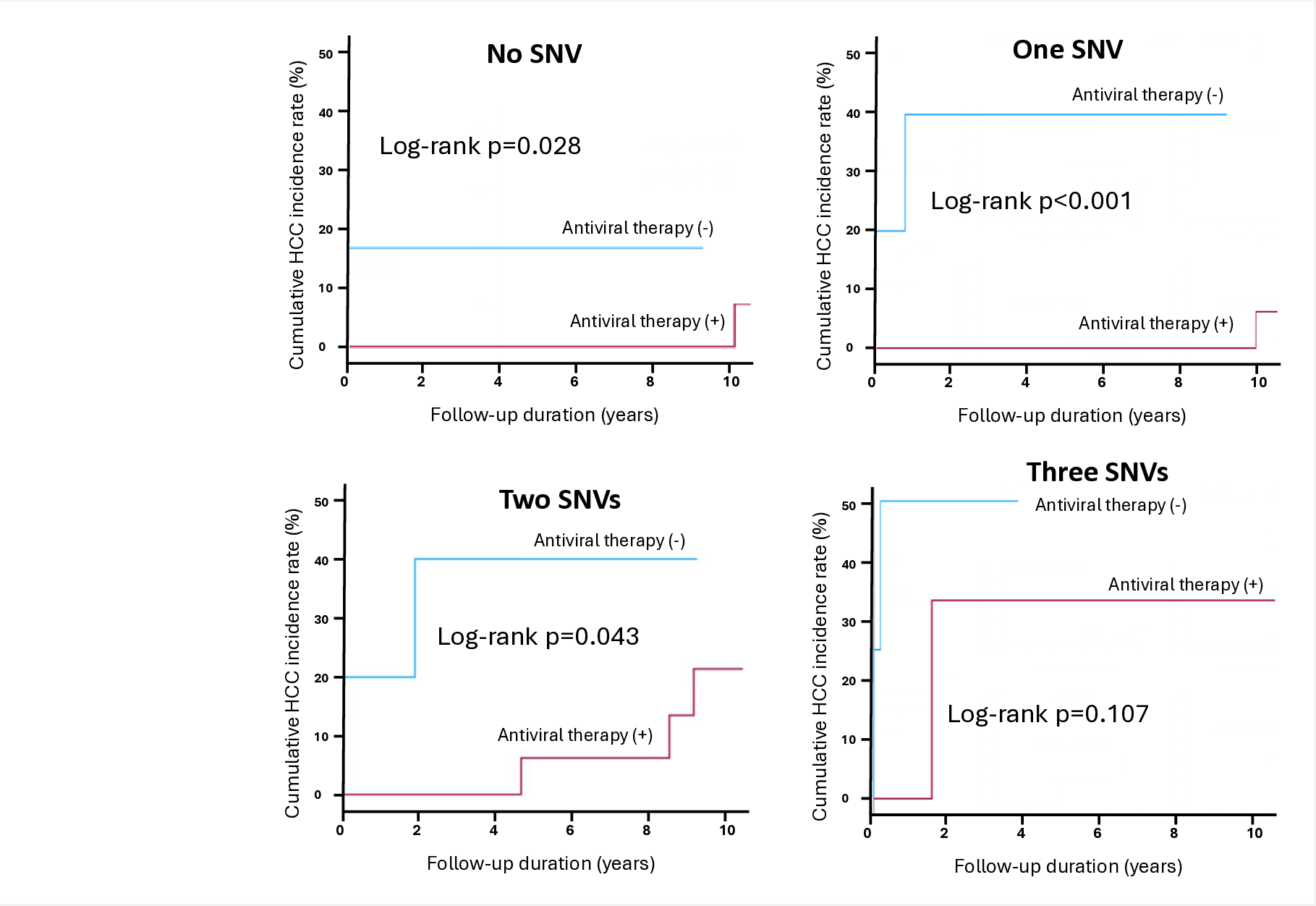

Supplement: Supplementary file 5 — Additional file 5. [file 12929_2025_1195_MOESM5_ESM.tiff]

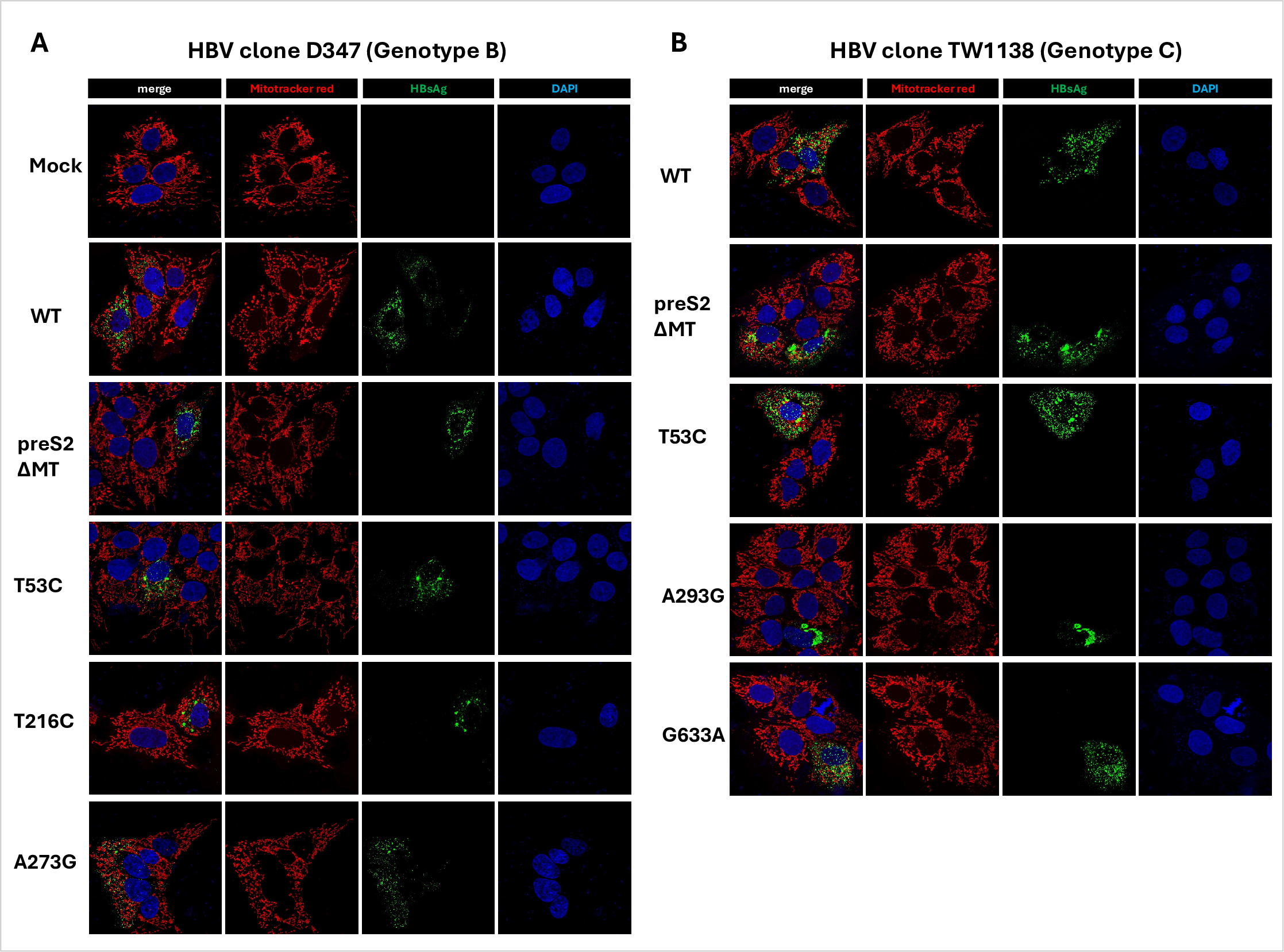

Supplement: Supplementary file 6 — Additional file 6. [file 12929_2025_1195_MOESM6_ESM.tif]

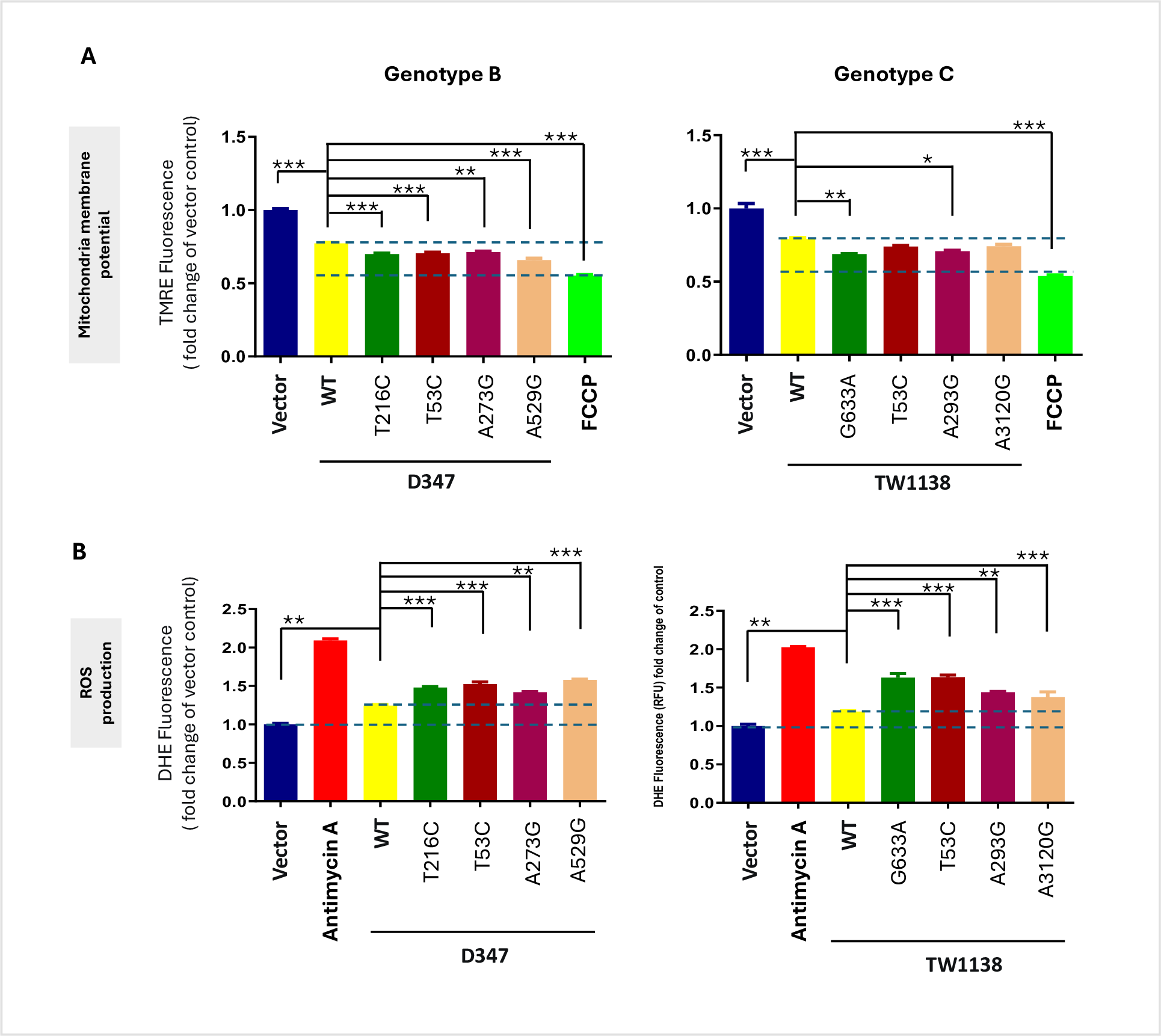

Supplement: Supplementary file 7 — Additional file 7. [file 12929_2025_1195_MOESM7_ESM.tif]

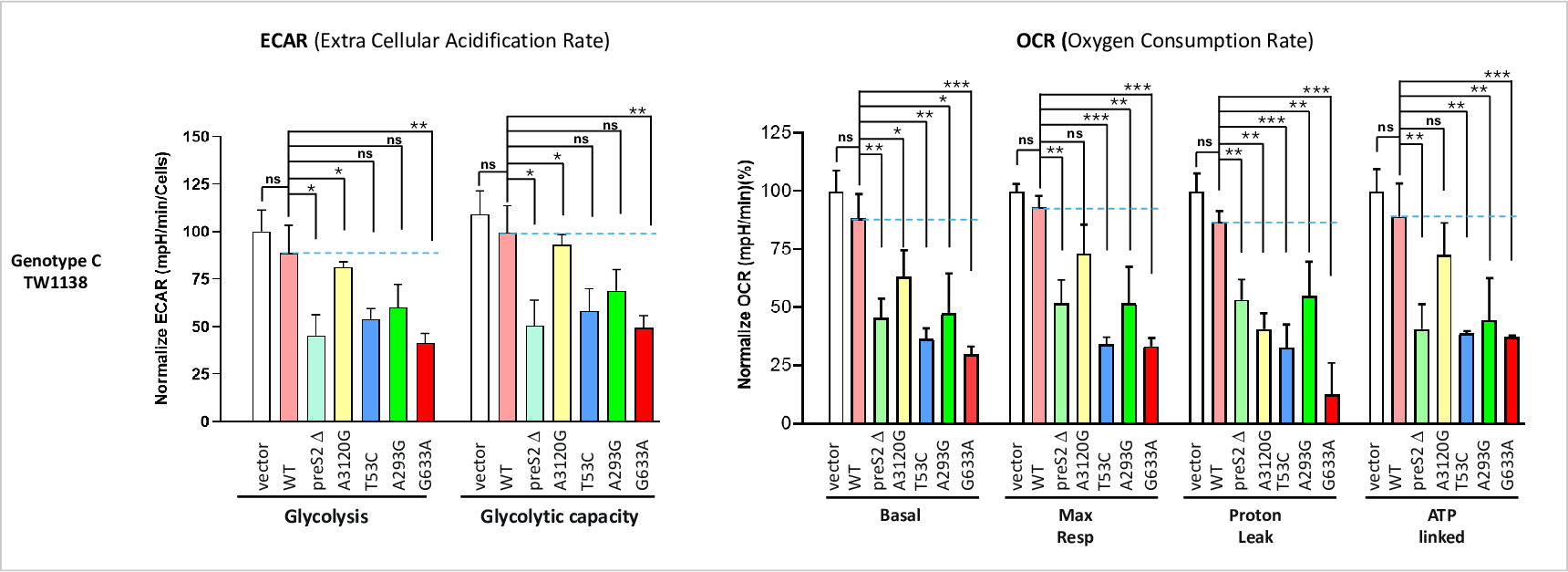

Supplement: Supplementary file 8 — Additional file 8. [file 12929_2025_1195_MOESM8_ESM.tif]

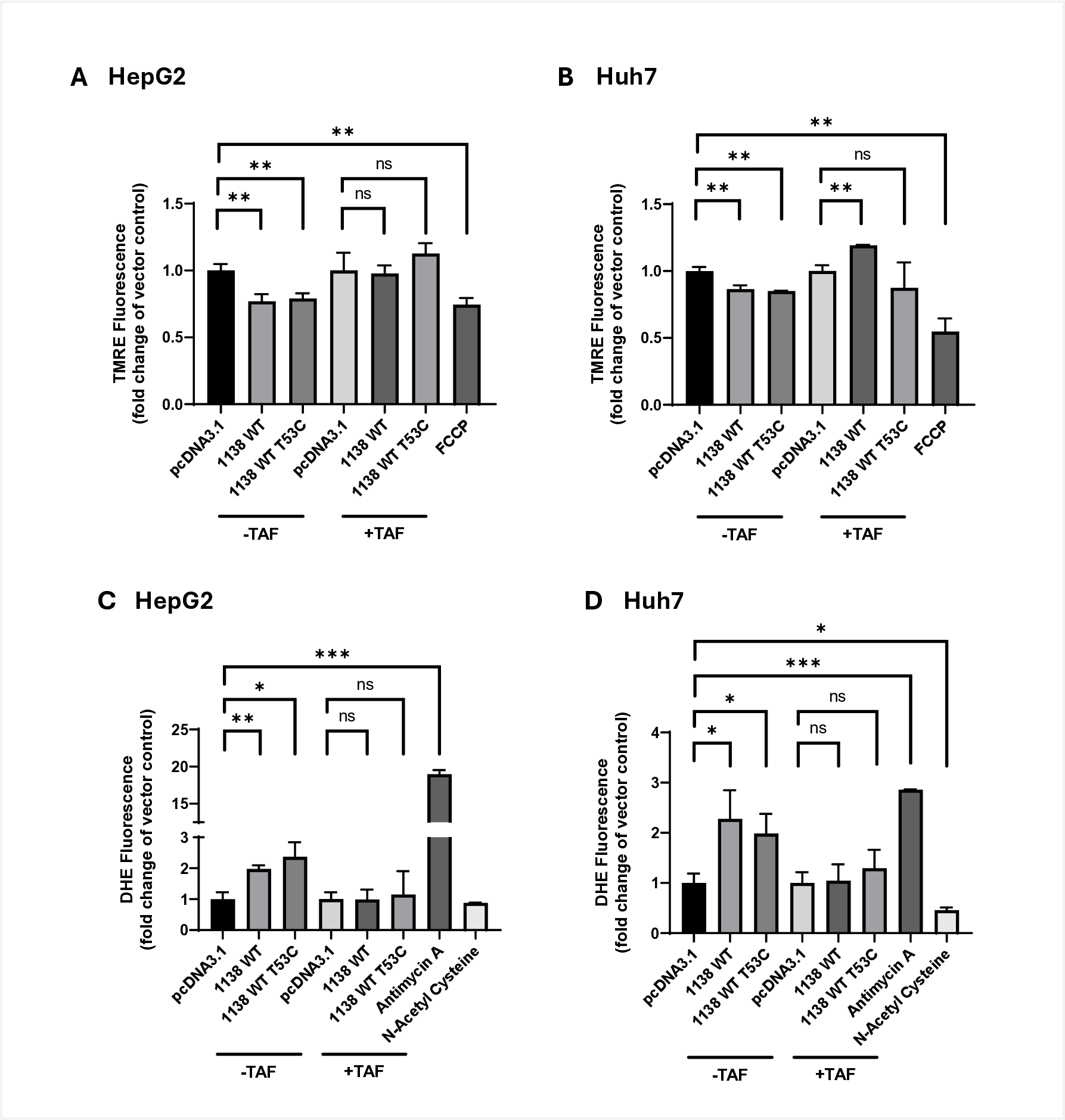

Supplement: Supplementary file 9 — Additional file 9. [file 12929_2025_1195_MOESM9_ESM.tiff]
