# Supplementary material for: The impact of single-nucleotide variants of hepatitis B virus and antiviral on liver cancer in gray zone patients
Source: J Biomed Sci. 2025 Dec 1;32:101. doi: 10.1186/s12929-025-01195-x (PMC12667080; doi:10.1186/s12929-025-01195-x)
Supplement: Supplementary file 10 — Additional file 10. [file 12929_2025_1195_MOESM10_ESM.docx]

**Supplementary Information**

**The impact of single-nucleotide variants of Hepatitis B virus and antiviral on liver cancer in gray zone patients**

Wei Teng^1,2#^, Ting-Tsung Chang^3#^, Chien-Wei Su^4,5^, Jun-Hao Xu^1^, Chiu-Chi Hsu^1^, Yu-Cheng Chang^1^, Wen-Chun Liu^6^, Yu-Wei Chiou^1,7^, Yu-Chuan Chang^1,7^, Yuh-Jin Liang^1,7,8*^, Jaw-Ching Wu^1,7,8*^

**Table of contents**

Supplementary Materials and Method2

Supplementary Figure legends12

Supplementary Videos legends 16

Supplementary Tables list17

Supplementary Tables18

References27

**Supplementary Materials and Method**

**Patient enrollment**

This was a retrospective study of 104 consecutive CHB patients in the gray zone who were serum hepatitis B surface antigen (HBsAg) positive and HCC-naïve for more than 6 months before enrollment. They were mainly followed by Prof. Jaw-Ching Wu at Taipei Veterans General Hospital and Prof. Ting-Tsung Chang at National Cheng Kung University Hospital between 1994 and 2014. All serum samples were obtained before antiviral treatment, and HBV genomes in sera were successfully amplified by polymerase chain reaction (PCR) and sequenced by direct sequencing or next generation sequencing (NGS). All patients tested negative for hepatitis C virus, hepatitis D virus, and human immunodeficiency virus serum markers. CHB patients in the gray zone of current treatment guidelines were defined as having serum ALT < 2 times the upper limit of normal (ULN) or HBV-DNA < 2000 IU/ml persistently and thus were not eligible for reimbursement of antiviral treatment in follow-up clinics for at least 2 years. Patients with both ALT ≥ 2 ULN and HBV DNA ≥ 2000 IU/ml were excluded. Patients with cirrhosis diagnosed based on histological findings by liver biopsy and/or ultrasonographic findings and/or the use of transient elastography and/or endoscopic findings of cirrhosis (varices, portal gastropathy) were also excluded. Finally, 104 patients in the gray zone were enrolled in the analysis (**Supplementary Figure 1**). In our previous study(1), the percentage of SNVs clones that accounted for ≥ 20% of the total viral populations was significantly associated with HCC using next-generation sequencing, sequencing of multiple clones, or direct sequencing (detection limit of 20%). In this longitudinal study, we also used HCC-associated SNVs, accounting for 20% of viral populations at baseline of enrollment, as a cutoff value for the prediction of HCC development during the follow-up period. The study was approved by the Institutional Review Board, Taipei Veterans General Hospital, Taiwan. (2021-02-013BC; 2021-09-016ACF).

**Clinical evaluation and follow-up**

Within 10 years of the study, all patients underwent routine blood chemistry testing, serum HBV DNA levels, other viral markers, ultrasonography, and assays of serum alpha-fetoprotein (AFP) levels every 6 months. HCC was diagnosed based on typical sonographic, dynamic computed tomography (CT), and/or magnetic resonance imaging (MRI)(2). Some enrolled patients who did not meet the current reimbursed-treatment guidelines and received antiviral therapy during follow-up included those who participated in a clinical trial or those who agreed to receive self-paid antiviral therapy due to the concern of progression to cirrhosis or HCC. The initial nucleos(t)ide analogues (NA) included entecavir (ETV), lamivudine (LAM), telbivudine (Ldt), adefovir (ADV), tenofovir disoproxil fumarate (TDF) and tenofovir alafenamide (TAF) monotherapy once tablet daily.

**Risk score construction**

This scoring system was adopted from our previous study (3), which identified four independent risk factors for HCC in “gray zone” CHB patients and assigned point values to each factor. Specifically, older age carries the greatest weight (age ≥ 50 years = 5 points; age 40–49 = 1 point; age < 40 = 0), male sex contributes 3 points (female = 0), a family history of HCC contributes 5 points (none = 0), and HBV DNA ≥ 2000 IU/mL contributes 1 point (HBV DNA < 2000 IU/mL = 0). The total risk score ranges up to 14 points, and the threshold was determined through ROC analysis to optimally balance sensitivity and specificity for HCC prediction. The cut-off of 8 was chosen by ROC curve analysis in our cohorts, aiming for the maximal Youden index (sensitivity + specificity – 1). A score of 8 points was found to best discriminate high-risk patients: almost all patients with scores <8 remained HCC-free at 3, 5, and 10 years of follow-up, whereas patients with ≥8 points had markedly higher cumulative HCC incidence. This risk score system, based on hospital cohorts from six medical centers, has been externally validated by the REVEAL community cohort (3).

**Quantitative analysis of HBsAg (HBV surface antigen)**

Serum HBsAg levels were quantified using the Architect HBsAg QT assay (Abbott Diagnostics, Germany). In brief, samples were mixed with anti-HBs-coated paramagnetic beads, washed, added with acridinium-labeled anti-HBs conjugate and reactant, washed, and added with pre-trigger and trigger solutions. Resulting chemiluminescence (proportional to HBsAg concentration with a lower limit of detection of 0.05 IU/mL) was measured. HBsAg titers in the serum were quantified according to the manufacturer's instructions.

**Measurement and sequencing of biochemical and serological markers in serum**

Hepatitis B e antigen (HBeAg) and antibodies against HBeAg (anti-HBe) were tested using an enzyme-linked immunosorbent assay kit (Abbott Laboratories, North Chicago, IL, USA). Antibodies against HDV (DiaSorin, Saluggia, Italy) were tested using an enzyme immunoassay kit, whereas anti-HCV antibodies were assessed using a second-generation enzyme immunoassay kit (Abbott Laboratories, North Chicago, IL, USA). Serum biochemistry was measured using the Roche/Hitachi Modular Analytics Systems (Roche Diagnostics GmbH, Mannheim, Germany), and serum AFP levels were tested by radioimmunoassay (Serono Diagnostic SA, Coinsin/VD, Switzerland).

**Detection and quantification of HBV DNA, HBV genotyping and sequencing in serum**

The complete HBV envelope open-reading frame was amplified using a pair of primers B2822(+) (5’-ggg,TCA,CCA,-TAT,TCT,Tgg-3’) and B840R (5’-

ACC,CCA,TCT,TTT,TgT, TTT, gTT,Agg-3’) that covered the entire coding region of the preS1, preS2, and S gene (2848-3215 and 1-835) via polymerase chain reaction (PCR). The PCR was performed in a thermal cycler (Perkin Elmer Cetus Corp., Norwalk, CT), beginning at 95℃ for 5 min, followed by 35 cycles (each cycle: 95℃ for 40 sec, 55℃ for 40 sec, 72℃ for 2 min) of amplification, and ending at 72℃ for 10 min. The PCR products were analyzed in 1.5% agarose gel, followed by ethidium bromide staining. The amplified PCR products were ligated into the plasmid pCR2 vector (Original TA cloning Kit; Invitrogen Corporation, Carlsbad, CA, USA) according to the manufacturer’s instructions. The ligation mixture was used to transform competent Escherichia coli strain DH5a (Gibco BRL, Life Technologies, Gaithersburg, MD, USA) and incubated overnight in LB agar plates containing 100 mg/ml ampicillin and 30 mg/ml X-gal at 37 °C. Successful ligation clones in blue white screening were picked and cultured overnight in 3 ml LB-Amp broth at 37 °C. Plasmid DNA was purified using a QIAprep Spin MiniPrep Kit (QIAGEN GmbH, D-40724 Hilden). Sequencing of the preS1, preS2, and S genes was performed using a dye terminator cycle sequencing kit (Dye Terminator Cycle Sequencing Core Kit #402117, Perkin Elmer Cetus Corp., Norwalk, CT) according to the manufacturer’s instructions, and sequencing products were analyzed using an ABI 373A sequencer (Perkin Elmer Cetus Corp., Norwalk, CT, USA). HBV DNA levels were measured using a Cobas Amplicor HBV monitor (Roche Diagnostic System, Basel, Switzerland) with a detection limit of 10 IU/ml. HBV genotyping was performed by PCR restriction fragment length polymorphism (PCR-RFLP) of the HBV surface gene and further verified by sequencing, as previously described(4).

**Generation of HBV SNVs DNA Plasmids**

HBV whole genome clones TW1138 (genotype C; GenBank #EF494377.1) and D347 (genotype B; GenBank #EF494382.1) were obtained by PCR amplification of patient serum samples. The 1.34mer replication-competent HBV DNA plasmid was generated using a previously described strategy(5, 6) and cloned into the pcDNA3.1 vector. To construct HBV whole-genome plasmids carrying SNVs, site-directed mutagenesis was performed using the NEBuilder HiFi DNA Assembly Master Mix (New England Biolabs), with complementary mutations introduced in the primers at the overlap region. Primers used for site-directed mutagenesis of the preS/S region of TW1138 and D347 are shown in **Supplementary Figure 2** and **Supplementary Figure 3**, respectively. The nucleotide sequences of these SNV constructs were confirmed by Sanger sequencing, using primers upstream of the SNV regions.

**Cell Culture**

Huh7 cells were maintained in DMEM (Life Technologies, Grand Island, NY, USA) supplemented with 10% fetal bovine serum (FBS). Transfection was performed on Huh7 cells using the Lipofectamine 3000 transfection reagent (Thermo Fisher Scientific; St. Louis, MO, USA).

**MitoTracker Red staining**

Live cells were grown on 35-mm glass-bottom dishes (Ibidi) and incubated in

medium containing 200 nM MitoTracker Red (M7512; Invitrogen) for 15 min at 37

°C under a 5% CO2 atmosphere. Cell images were collected every minute for

time-lapse imaging. At each time point, 20 z-series optical slices were obtained (step

1.0 mm) using a confocal fluorescence microscope (LSM 880 with AiryScan).

Zeiss; Oberkochen, Germany) and the ZEN BLUE imaging software. For co-staining

with HBsAg, cells were fixed with 1% paraformaldehyde, permeabilized with 1%

(v/v) NP-40 in PBS and stained with an anti-HBsAg antibody (cat # sc-53299; Santa Cruz). Confocal microscopy images were obtained using an Olympus FV10i Confocal Fluorescence Microscope (Waltham, MA, USA).

**Immunofluorescence staining**

Cells were grown on glass cover slips, fixed in 4% paraformaldehyde in PBS

for 15 min at room temperature (RT) and permeabilized with 0.5% Triton X-100 in PBS for 5 min. Fixed cells were blocked with 5% BSA in PBS for 30 min, incubated overnight at 4 °C with the primary antibody, washed, incubated for 1 h at RT with the secondary Ab, and counterstained with DAPI (Pharmingen). Cover slips were mounted with glycerol mounting medium (Dako) and sealed with clear nail polish. Fluorescence images were obtained using confocal immunofluorescence microscopy. The primary antibody used for immunofluorescence staining was mouse anti-HBsAg (clone 1023, cat # sc-53299; Santa Cruz). The secondary antibodies used were Alexa555-conjugated donkey anti-mouse IgG (Invitrogen) and Alexa488-conjugated donkey anti-rabbit IgG (Invitrogen).

**Seahorse Extracellular Flux Analysis**

Huh7 cells were transfected with the HBV expression plasmids or control vectors. After 48 hours, the cells were seeded into XFe24 Cell Culture Microplates (Agilent, cat. 100777-004) at a density of 3-4 x 10^4^ cells per well. Upon cell reaching 95% confluence, the medium was replaced with unbuffered Seahorse XF Base Medium (Agilent, cat. 103757-100) supplemented with 10 mM glucose, 2 mM L-glutamine, and 1 mM sodium pyruvate, and equilibrated for 1 hour at 37°C without CO2. The XF24 sensor cartridges were pre-hydrated overnight. On the next day, specific metabolic regulators were loaded: for the Mito-Stress test (Agilent, cat. 103015-100), 1.5 μM oligomycin, 1 μM FCCP, and 0.5 μM rotenone/antimycin A; for the Real-Time ATP Rate Assay Kit (Agilent, cat. 103591-100), 1.5 μM oligomycin and 0.5 μM rotenone/antimycin A; and for the Glycolysis Stress Test (Agilent, 103020-100), 10 mM glucose, 1.5 μM oligomycin, and 50 mM 2-DG. The sensor cartridges and cell culture microplates were assembled and analyzed using an XFe24 Analyzer with a built-in assay protocol. Immediately after each run, the cells plated on XFe24 plates were lysed in RIPA buffer, and the total protein concentration was determined using the BCA assay for normalization. The data were processed using the respective Agilent Report Generators, and statistical analysis was performed using Prism software (version 7.0; GraphPad).

**Mitochondrial membrane potential assay**

Tetramethylrhodamine (ethyl ester), a lipidophilic and positively charged fluorescent probe, easily accumulates within active mitochondria due to their negative surface charges. The TMRE-Mitochondrial Membrane Potential Assay Kit (Abcam, cat. ab113852) was used to assess mitochondrial membrane potential in cells expressing HBV SNVs. For TMRE staining, 200 nM Tetramethylrhodamine was added to the cells for 30 minutes at 37°C in darkness. To serve as a positive control for disrupted membrane potential, 20 µM carbonyl cyanide 4-(trifluoromethoxy) phenyl hydrazone (FCCP) was added to the staining solution for 10 min. Total fluorescence, representing the mitochondrial membrane potential, was measured using microplate spectrophotometry at Ex/Em 549/575 nm.

**ROS production assay**

Dihydroethidium (DHE) is a fluorescent probe specific to superoxide and hydrogen peroxide for the detection of ROS generation. DHE Assay Kit (Abcam, cat. ab236206) was used to measure the ROS levels in cells expressing HBV SNVs. For ROS staining, 5 µM Dihydroethidium in Cell-Based Assay Buffer was added to the cells for 30 minutes at 37°C in darkness. To serve as a positive control for blocking complex III of the mitochondrial electron transport chain, 10 µM Antimycin A used as the positive control for ROS generation. N-acetyl Cysteine (300 mM) was used as the antioxidant control. Total fluorescence, representing ROS production, was measured using microplate spectrophotometry at excitation at 480-520 nm and emission at 570-600 nm.

**Cytosolic Calcium Levels Measurement**

Huh7 cells were seeded onto a 25 mm glass bottom dish (Alpha Plus, cat. 16235-1S15) at a density of 1.2. x 10^5^ cells. After 24 h, HBV expression plasmids or virus-free control vectors were transfected into cells. Forty-eight hours post-transfection, intracellular Ca^2+^ levels were measured. Cells were washed twice with calcium-containing loading buffer (140 mM NaCl, 5 mM KCl, 10 mM HEPES, 1 mM MgCl2, 10 mM glucose, 2.2 mM CaCl2). They were then loaded with 3 µM of the Ca^2+^ indicator dye Fura-2 AM in the same buffer and incubated for 25 minutes at 37°C, protected from light. The cells were then washed three times with loading buffer and incubated for an additional 15 minutes to allow the hydrolysis of the ester group. The glass bottom dish was then mounted on the stage of a Zeiss Axio Observer D1 inverted microscope equipped with a 40× oil immersion objective (Fluar 40x/1.30 Oil M27) and an Electron-Multiplying CCD Evolve 512 camera.

To identify the transfected cells, co-transfection with an RFP expression plasmid was employed. This allowed for differentiation between transfected and non-transfected cells, as RFP expression does not interfere with Fura2-based measurements. Cytosolic calcium signals were triggered by addition of 100 µM ATP. Changes in calcium levels were monitored by alternately exciting the dye at 340 nm and 380 nm and recording the emission at 510 nm using MetaFluor fluorescence ratio imaging software. Fura-2 fluorescence images were acquired every 0.5 seconds for at total of 300 seconds. Before ATP addition, the F340/F380 ratio obtained in the cell-free area was recorded as the background, and the subsequent F340/F380 ratios were adjusted by subtracting this background value. After background correction, the F340/F380 ratio was used as a measure of cytosolic Ca^2+^ concentration.

**Statistical analysis**

Descriptive data with normal distribution are reported as mean ± standard deviation or as percentages; otherwise, they are presented as median (interquartile range, IQR). We used the independent Student’s t-test and Mann–Whitney U test to assess differences between groups for variables that showed normal and abnormal distributions, respectively. The chi-square test was used to assess differences between the two groups for categorical variables. The significant cutoff value for each specific SNV frequency was 20%, which could be detected by direct sequencing. A two-tailed P value of < 0.05 was considered statistically significant. Disease-free survival (DFS) was defined as the time from the date of enrollment or initial antiviral therapy until HCC occurrence was confirmed by imaging. Survival curves were calculated using the Kaplan–Meier method and compared using the log-rank test.

To investigate the association between antiviral therapy and the risk of developing HCC, a time-dependent Cox proportional hazards model was constructed. Because some patients initiated antiviral treatment after cohort enrollment not due to changes in HBV DNA levels or liver function, but instead as part of clinical trial participation or due to personal concerns about HCC risk, antiviral therapy use was treated as a time-varying covariate. Patients were considered unexposed until the date of antiviral therapy initiation and exposed thereafter. The time scale was defined from the date of study entry to the first diagnosis of HCC, death, or censoring at last follow-up. To select the most relevant predictors for HCC development, we applied a least absolute shrinkage and selection operator (LASSO) regression model based on the Cox proportional hazards framework. The LASSO method performs regularization and variable selection by imposing a penalty on the absolute size of regression coefficients. Ten-fold cross-validation was used to determine the optimal penalty parameter (λ) that minimized the partial likelihood deviance. Variables selected by LASSO were subsequently entered into a multivariable Cox regression model, where antiviral therapy use was modeled as a time-dependent covariate to account for its initiation during follow-up. Statistical analyses of patient studies were performed using SAS, version 9.4, and SPSS, version 20.0, software (SPSS, Chicago, IL).

**Supplementary Figure legends**

**Supplementary Figure 1. Flowchart of patients’ enrollment**

**Supplementary Figure 2. Primers and sequence information for SNVs plasmid construction in genotype C clone TW1138.** Site-directed mutagenesis was performed using the NEBuilder HiFi DNA Assembly Master Mix to construct HBV whole-genome plasmids carrying SNVs. Mutations (blue squares) were introduced using complementary primers in overlapping regions. Primers for site-specific mutagenesis are indicated by black arrow lines, showing the 3’ to 5’ direction. The start codons for preS2, preS1, and S (methionine) are marked in green.

**Supplementary Figure 3. Primers and sequence information for SNVs plasmid construction in genotype B clone D347.** Primers for site-specific mutagenesis are indicated by black arrow lines, showing the 3’ to 5’ direction. The start codons for preS2, preS1, and S (methionine) are marked in green.

**Supplementary Figure 4. Cumulative HCC incidence in CHB patients in the gray zone of the current treatment guidelines and effect of antiviral therapy. (A)** The 3-,5- and 10-year cumulative HCC incidences were 7%, 9%, and 14%, respectively. **(B)** Patients with HCC-associated-SNVs had higher cumulative HCC incidence (log-rank p=0.037). **(C)**Antiviral therapy significantly decreased HCC risk (log-rank p < 0.001). **(D)** Antiviral therapy significantly decreased HCC risk in patients with HCC-associated-SNVs (log-rank p < 0.001).

**Supplementary Figure 5. Effect of antiviral therapy in patients with and without HCC-associated SNVs in the gray zone.** Antiviral therapy may decrease HCC risk, although it did not reach statistical significance in the presence of all three HCC-associated SNVs.

**Supplementary Figure 6. Co-staining with MitoTracker Red and anti-HBs antibodies to assess the impact of SNVs expression on mitochondrial biogenesis.** Huh7 cells were transfected with (A) D347-WT, -preS2ΔMT, -T53C, -T216C, and -A273G and (B) TW1138-WT, -preS2ΔMT, -T53C, -A273G, and -G633A expression plasmids. Two days post-transfection, the cells were labeled with MitoTracker Red, immunostained with anti-HBsAg antibody (green), and counterstained with DAPI (blue). Compared with the no-virus vector control, cells expressing HBV, including WT, preS2ΔMT, and SNVs, displayed varying reductions in MitoTracker Red signal intensity and fragmented mitochondrial morphology.

**Supplementary Figure 7. Effect of HBV SNVs on mitochondrial membrane potential and ROS production.** HBV expression plasmids, including WT, preS2ΔMT, and SNVs of D347 and TW1138 were transfected into Huh7 cells for 48 hours. (A) Mitochondrial membrane potential was analyzed by measuring the fluorescence intensity of TMRE. Carbonyl cyanide m-chlorophenyl hydrazone (CCCP) was used as the depolarization control. (B) ROS production was analyzed by measuring the fluorescence intensity of DHE. Antimycin A, a known ROS generator, was used as the positive control. All data were obtained from at least three independent experiments. *p < 0.05, **p < 0.01, ***p < 0.001.

**Supplementary Figure 8. Effect of** **the TW1138-derivated SNVs on cell metabolism.** Huh7 cells were transfected with TW1138-WT and SNVs expression plasmids for 48 hours. OCR and ECAR were measured using the Seahorse XF96 instrument with the Glycolysis Stress Test and Mito Stress Test, respectively. TW1138-derivated SNVs had a significant impact on OCR and a minor effect on ECAR. Statistical analysis was performed using one-way ANOVA in GraphPad Prism (*p < 0.05; **p < 0.01; ***p < 0.001) (N=3-4).

**Supplementary Figure S9. HBV-induced mitochondrial dysfunction in HepG2 and Huh7 cells and the protective effect of tenofovir alafenamide (TAF).** (A, B) Mitochondrial membrane potential was assessed in HepG2 (A) and Huh7 (B) cells transfected with vector control, wild-type HBV plasmid (1138WT), or the T53C single-nucleotide variant (1138-T53C) for 72 hours. Both HBV constructs significantly reduced mitochondrial membrane potential compared to the vector control (pcDNA3.1). Treatment with tenofovir alafenamide (TAF, 800 nM, administered at the time of transfection) effectively attenuated these effects. CCCP was used as a positive control for mitochondrial depolarization. (C, D) Intracellular reactive oxygen species (ROS) levels were measured in HepG2 (C) and Huh7 (D) cells under the same experimental conditions. Both HBV constructs significantly increased ROS production compared to the vector control, and these effects were mitigated by TAF treatment. Antimycin A, a known ROS inducer, was used as a positive control, while N-acetyl cysteine (NAC), a ROS scavenger, served as a negative control. Data are representative of three independent experiments. Error bars indicate standard deviation (n = 3). Statistical significance was determined by one-way ANOVA followed by Tukey’s multiple comparisons test; *p < 0.05, **p < 0.01, ***p < 0.001.

**Supplementary Videos legends**

**Supplementary Video 1.** **Mitochondrial dynamics in Huh7 cells transfected with** **the pCDNA vector control.** Huh7 cells were transfected with the pCDNA vector, labeled with MitoTracker Red, and imaged. Time-lapse images were acquired using fluorescence live-cell confocal microscopy for 50 frames with 2-min increment.

**Supplementary Video 2.** **Mitochondrial dynamics in Huh7 cells transfected with the genotype C HBV clone TW1138-WT.**

**Supplementary Video 3.** **Mitochondrial dynamics in Huh7 cells transfected with preS2ΔMT TW1138.**

**Supplementary Video 4.** **Mitochondrial dynamics in Huh7 cells transfected with TW1138-A293G.**

**Supplementary Video 5.** **Mitochondrial dynamics in Huh7 cells transfected with TW1138-G633A.**

**Supplementary Video 6.** **Mitochondrial dynamics in Huh7 cells transfected with TW1138-T53C.**

**Supplementary Video 7.** **Mitochondrial dynamics in Huh7 cells transfected with the genotype B HBV clone D347-WT.**

**Supplementary Video 8.** **Mitochondrial dynamics in Huh7 cells transfected with D347-T216C.**

**Supplementary Video 9.** **Mitochondrial dynamics of Huh7 cells transfected with D347-A273G.**

**Supplementary Tables list**

**Supplementary Table 1. HBV treatment recommendation for non-cirrhosis patients based on major organization guidelines**

**Supplementary Table 2. Baseline characteristics of patients in the gray zone with genotype B or C HBV**

**Supplementary Table 3. Six single-nucleotide variants (SNVs) in Pre-S/S region between patients with HCC vs. HCC-free among genotype B hepatitis B virus (HBV)-infected patients in the gray zone**

**Supplementary Table 4. Twenty-one single-nucleotide variants (SNVs) in Pre-S/S region between patients with HCC vs. HCC-free among genotype C hepatitis B virus (HBV)-infected patients in the gray zone**

**Supplementary Table 5. Patients with candidate HCC-associated SNVs in the gray zone**

**Supplementary Table 6. Baseline characteristics of 15 HCC patients in the gray zone of current treatment guideline**

**Supplementary Table 1**. HBV treatment recommendation for non-cirrhosis patients based on major organization guidelines

| **Guidelines** | **HBeAg positive** | | | **HBeAg negative** | | |
| --- | --- | --- | --- | --- | --- | --- |
|  | **HBV DNA (IU/ml)** | **ALT**  **(U/L)** | **Histology** | **HBV DNA (IU/ml)** | **ALT**  **(U/L)** | **Histology** |
| **APASL (2015)** | >20,000 | >2×ULN**^*^** | >ULN and  Ishak ≥ F3/ METAVIR ≥F2 by biopsy | >2,000 | >2×ULN**^*^** | >ULN and  Ishak ≥ F3/ METAVIR ≥F2 by biopsy |
| **AASLD (2018)** | >20,000 | >2×ULN**^#^** | >ULN and  METAVIR ≥F2 or ≥A3 by biopsy | >2,000 | >2×ULN**^#^** | >ULN and  METAVIR ≥F2 or ≥A3 by biopsy |
| **EASL (2025)** | >2,000 | >ULN**^*^** | Ishak ≥F3/  METAVIR ≥F2 | >2,000 | >ULN**^*^** | Ishak ≥F3/  METAVIR ≥F2 |
| **WHO**  **(2024)** | **Recommendations for treatment**  Treatment for all adults and adolescents (aged ≥12 years) with chronic hepatitis B (CHB) (including pregnant women and girls and women of reproductive age) with:   1. Significant fibrosis (≥F2) based on an APRI score of >0.5 or transient elastography value of >7 kPa or cirrhosis (F4) based on clinical criteria (or an APRI score of >1 or transient elastography value of >12.5 kPa), regardless of HBV DNA or ALT levels. (adults: strong recommendation, moderate-certainty evidence; adolescents: strong recommendation, low-certainty evidence) 2. HBV DNA >2000 IU/mL and an ALT level above the upper limit of normal (ULN) (30 U/L for men and boys and 19 U/L for women and girls). For adolescents, this should be based on ALT>ULN on at least two occasions in a 6- to 12-month period (adults: strong recommendation, high-certainty evidence [HBV DNA >20,000 IU/mL] and low-certainty evidence [HBV DNA 2000–20,000 IU/mL]; adolescents: conditional recommendation, low-certainty evidence) 3. Coinfections (such as HIV, hepatitis D or hepatitis C); family history of liver cancer or cirrhosis; immune suppression (such as long-term steroid use, solid organ or stem cell transplant); comorbidities (such as diabetes or metabolic dysfunction–associated steatotic liver disease); or extrahepatic manifestations (such as glomerulonephritis or vasculitis), regardless of the APRI score or HBV DNA or ALT levels. (adults: strong recommendation, moderate-certainty evidence; adolescents: conditional recommendation, low-certainty evidence)   In the absence of access to an HBV DNA assay: Persistently abnormal ALT levels alone (defined as two ALT values above the ULN at unspecified intervals during a 6- to 12-month period), regardless of APRI score. (adults and adolescents: conditional recommendation, very-low certainty eviden*ce* | | | | | |

Abbreviations: ALT, alanine aminotransferase; HCC, hepatocellular carcinoma; LSM, liver stiffness measurement; ULN, upper limit of normal

***** ALT ULN：40 U/L for Asian-Pacific Association for the Study of the Liver (APASL) and European Association for the Study of the Liver (EASL)

**#** ALT ULN：35 U/L for men and 25 U/L for women for American Association for the Study of the Liver (AASLD)

APRI score, AST to Platelet Ratio Index

APASL guidelines usually require 3 months to observe if there is persistently ALT>2x ULN. If the ALT levels <2x ULN, guidelines require monitoring every 3 months to observe if ALT>ULN, severe inflammation or moderate liver fibrosis by biopsy, age>35 years old in the presence of HCC family history.

AASLD guideline: Requires 3 months monitoring to evaluate if there is persistently elevated ALT>2xULN. For ALT<2xULN, guidelines also require monitoring every 3 months, to treat if noninvasive tests show ≧F2 fibrosis, serum HBV DNA>2000 IU/ml, esp. >40 years old. HCC family history can be treated.

EASL Guidelines: Risk factors, such as family history should be taken into the indication of antiviral therapy regard less of HBV DNA, ALT levels, or fibrosis stage.

| **Variables** | **Genotype B (n=75)** | | **Genotype C (n=29)** | **P-value** | | |
| --- | --- | --- | --- | --- | --- | --- |
| **Demographics** |  |  | | |  |  |
| Age (year-old) | 48.5 (IQR 38.7-57.9) | 46.2 (IQR 41.0-62.9) | | | 0.951 |  |
| Male gender | 51 (68.0) | 14 (48.3) | | | 0.062 |  |
| HBeAg positive* | 20/71 (28.2) | 15/25 (60.0) | | | 0.004 |  |
| Family history of HCC* | 18/66 (27.3) | 9/23 (39.1) | | | 0.287 |  |
| Hypertension, n (%) | 14 (18.7) | 5 (17.2) | | | 0.609 |  |
| Diabetes mellitus, n (%) | 5 (6.7) | 1 (3.4) | | | 0.498 |  |
| Alcohol use, n (%) | 3 (4.0) | 1 (3.4) | | | 0.452 |  |
| Fatty liver, n (%) | 30 (40.0) | 13 (44.8) | | | 0.672 |  |
| Immunosuppressants use, n (%) | 1 (1.3) | 1 (3.4) | | | 0.651 |  |
| FIB-4 score | 1.00 (IQR 0.60-1.60) | 1.36 (IQR 0.73-2.32) | | | 0.049 |  |
| APRI score | 0.33 (IQR 0.22-0.47) | 0.50 (IQR 0.35-0.67) | | | 0.005 |  |
| Anti-viral therapy | 55 (73.3) | 28 (96.6) | | | 0.008 |  |
| **Biochemical factor** |  |  | | |  |  |
| Log HBV DNA (IU/ml) | 4.8 (IQR 3.9-6.2) | 7.1 (IQR 5.3-8.0) | | | 0.001 |  |
| qHBsAg > 250 (IU/ml), n (%)* | 36/45 (80.0) | 4/4 (100.0) | | | 0.322 |  |
| AST (IU/ml) | 29 (IQR 23-40) | 41 (IQR 32-55) | | | 0.001 |  |
| ALT (IU/ml) | 44 (IQR 32-60) | 57 (IQR 38-78) | | | 0.043 |  |
| Total-bilirubin (mg/dL) | 0.7 (IQR 0.5-0.9) | 0.7 (IQR 0.6-1.0) | | | 0.477 |  |
| Platelet (10^3^/μL) | 216 (IQR 173-259) | 207 (IQR 164-241) | | | 0.304 |  |
| Albumin (g/dL) | 4.5 (IQR 4.3-4.7) | 4.3 (IQR 4.1-4.6) | | | 0.018 |  |
| AFP (ng/mL) | 4.7 (IQR 3.2-6.9) | 6.1 (IQR 3.3-8.9) | | | 0.347 |  |

**Supplementary Table 2. Baseline characteristics of patients in the gray zone with genotype B or C HBV**

* Missing data at the time of enrollment for this parameter

Abbreviations: AFP, Alpha-fetoprotein; ALT, alanine aminotransferase; AST, aspartate aminotransferase; APRI score, AST to Platelet Ratio Index; FIB-4 score, Fibrosis index based on the 4 factors (age, platelet counts, ALT, AST) score; HBV, hepatitis B virus; HCC, hepatocellular carcinoma; qHBsAg, quantitative hepatitis B surface antigen

**Supplementary Table 3. Six single-nucleotide variants (SNVs) in Pre-S/S region between patients with HCC vs. HCC-free among genotype B hepatitis B virus (HBV)-infected patients in the gray zone**

| **Site** | **Nucleotides** | | **Amino acid** | **HCC (n=11)** | **HCC-free (n=58)** | **P-value** |
| --- | --- | --- | --- | --- | --- | --- |
|  | **Wild** | **Mutant** |  |  |  |  |
| 53 | T | C | F141L | 6 (54.5) | 17 (29.3) | 0.042 |
| 216 | T | C | L195S | 5 (45.5) | 20 (34.5) | 0.388 |
| 273 | A | G | N214S | 6 (54.5) | 18 (31.0) | 0.043 |
| 529 | A | G | T299T | 8 (72.7) | 26 (44.8) | 0.040 |
| 530 | G | A | I300T | 10 (90.9) | 55 (94.8) | 0.610 |
| 724 | T | C | V364V | 10 (90.9) | 56 (96.6) | 0.400 |

**Supplementary Table 4. Twenty-one single-nucleotide variants (SNVs) in Pre-S/S region between patients with HCC vs. HCC-free among genotype C hepatitis B virus (HBV)-infected patients in the gray zone**

| **Site** | **Nucleotides** | | **Amino acid** | **HCC (n=4)** | **HCC-free (n=25)** | **P-value** |
| --- | --- | --- | --- | --- | --- | --- |
|  | **Wild** | **Mutant** |  |  |  |  |
| 53 | T | C | F141L | 3 (75.0) | 9 (36.0) | 0.046 |
| 293 | A | G | T221A | 0 (0) | 2 (8.0) | 0.558 |
| 446 | C | G | L272V | 0 (0) | 2 (8.0) | 0.558 |
| 456 | A | G | Q275R | 0 (0) | 0 (0) | - |
| 633 | G | A | R334K | 1 (25.0) | 1 (4.0) | 0.044 |
| 834 | A | G | Stop codon | 1 (25.0) | 5 (20.0) | 0.819 |
| 2875 | C | A | Q10K | 0 (0) | 9 (36.0) | 0.098 |
| 2889 | A | G | T14T | 3 (75.0) | 17 (68.0) | 0.779 |
| 2901 | C | T | V18V | 4 (100) | 17 (68.0) | 0.094 |
| 2931 | T | C | H28H | 4 (100) | 17 (68.0) | 0.094 |
| 2988 | G | C | P47P | 4 (100) | 17 (68.0) | 0.094 |
| 2989 | C | A | H48N | 4 (100) | 17 (68.0) | 0.094 |
| 2997 | C | T | D50D | 4 (100) | 17 (68.0) | 0.094 |
| 2998 | A | C | N51H | 4 (100) | 17 (68.0) | 0.094 |
| 3006 | G | A | P53P | 4 (100) | 17 (68.0) | 0.094 |
| 3009 | C | G | D54E | 4 (100) | 17 (68.0) | 0.094 |
| 3016 | A | C | K57Q | 4 (100) | 17 (68.0) | 0.094 |
| 3021 | G | A | V58V | 4 (100) | 17 (68.0) | 0.094 |
| 3066 | A | T | E73D | 4 (100) | 16 (64.0) | 0.088 |
| 3097 | C | A | L84I | 4 (100) | 17 (68.0) | 0.094 |
| 3120 | A | G | A91A | 2 (50.0) | 7 (28.0) | 0.049 |

**Supplementary Table 5. Patients with candidate HCC-associated SNVs in the gray zone**

| **Variables** | **Anti-viral therapy** | | **P-value** |
| --- | --- | --- | --- |
|  | **Yes (n=49)** | **No (n=15)** |  |
| **Demographics** |  |  |  |
| Age (year-old) | 47.7 (IQR 39.3-59.4) | 51.7 (IQR 36.9-63.4) | 0.531 |
| Male gender | 31 (63.3) | 9 (60.0) | 0.819 |
| Genotype B/C | 34/15 (69.4/30.6) | 14/1 (93.3/6.7) | 0.061 |
| HBeAg positive* | 11/48 (22.9) | 1/12 (8.3) | 0.159 |
| Family history of HCC* | 15/48 (31.3) | 1/12 (8.3) | 0.098 |
| FIB-4 score | 1.10 (IQR 0.70-1.85) | 0.90 (IQR 0.70-1.80) | 0.913 |
| APRI score | 0.39 (IQR 0.24-0.56) | 0.26 (IQR 0.20-0.39) | 0.082 |
| HBeAg (+) or HBV DNA ≥ 2000 IU/ml | 42 (85.7) | 13 (86.7) | 0.926 |
| **Biochemical factor** |  |  |  |
| Log HBV DNA (IU/ml) | 4.9 (IQR 3.4-6.0) | 4.2 (IQR 3.1-6.2) | 0.585 |
| HBV ≥ 2000 IU/ml | 32 (65.3) | 8 (53.3) | 0.812 |
| AST (IU/ml) | 37 (IQR 26-49) | 23 (IQR 19-36) | 0.013 |
| ALT (IU/ml) | 50 (IQR 38-77) | 32 (IQR 23-40) | 0.001 |
| Total-bilirubin (mg/dL) | 0.7 (IQR 0.5-0.9) | 0.7 (IQR 0.5-1.2) | 0.648 |
| Platelet (10^3^/μL) | 193 (IQR 166-243) | 237 (IQR 181-285) | 0.187 |
| Albumin (g/dL) | 4.5 (IQR 4.3-4.6) | 4.4 (IQR 4.2-4.8) | 0.575 |
| AFP (ng/mL) | 5.1 (IQR 3.6-7.6) | 3.9 (IQR 2.6-7.7) | 0.198 |

* Missing data at the time of enrollment for this parameter

Abbreviations: AFP, Alpha-fetoprotein; ALT, alanine aminotransferase; AST, aspartate aminotransferase; APRI score, AST to Platelet Ratio Index; FIB-4 score, Fibrosis index based on the 4 factors (age, platelet counts, ALT, AST) score; HBV, hepatitis B virus; HCC, hepatocellular carcinoma; qHBsAg, quantitative hepatitis B surface antigen

**Supplementary Table 6. Baseline characteristics of 15 HCC patients in the gray zone of current APASL treatment guideline**

| **No** | **Age**  **(y/o)** | **Sex** | **ALT**  **(IU/ml)** | **HBV DNA (Log IU/ml)** | **FIB-4** | | | **APRI** | | **HCC-SNVs** | **HCC family**  **history** | **Anti-viral therapy** | | | **Outside HBV**  **treatment guideline** | | |
| --- | --- | --- | --- | --- | --- | --- | --- | --- | --- | --- | --- | --- | --- | --- | --- | --- | --- |
|  |  |  |  |  |  |  |  |  |  |  |  | **Use** | **Time to use**  **(months)** | **Treatment duration**  **(months)** | **AASLD**  **(2018)** | **EASL**  **(2025)** | **WHO**  **(2024)** |
|  |  |  |  |  | **Baseline** | **HCC** | **Baseline** | | **HCC** |  |  |  |  |  |  |  |  |
| 1 | 62.9 | M | 29 | 1.76 | 2.70  (F2) | 3.33  (≥ F3) | 0.57  (≥ F2) | | 0.51  (≥ F2) | **+** | **-** | **-** |  |  | **+** | **+** | **-** |
| 2 | 58.7 | M | 39 | 7.88 | 2.00  (F2) | 2.46  (F2) | 0.53  (≥ F2) | | 0.70  (≥ F2) | **+** | **+** | **-** |  |  | **-** | **-** | **-** |
| 3 | 55.0 | M | 55 | 3.88 | 5.70  (≥ F3) | 5.41  ≥ F3 | 1.93  (F4) | | 1.82  (F4) | **-** | **+** | **-** |  |  | **-** | **-** | **-** |
| 4 | 67.8 | F | 68 | 4.30 | 10.3  (≥ F3) | 4.50  (≥ F3) | 6.78  (F4) | | 0.73  (≥ F2) | **+** | **+** | **+** | 1.9 | 12.6 | **-** | **-** | **-** |
| 5 | 63.3 | M | 62 | 2.88 | 1.69  (F2) | 3.95  (≥ F3) | 0.53  (≥ F2) | | 1.18  (F4) | **+** | **-** | **-** |  |  | **+** | **+** | **-** |
| 6 | 66.4 | M | 24 | 3.42 | 1.70  (F2) | 1.99  (F2) | 0.31  (F0-1) | | 0.31  (F0-1) | **+** | **+** | **+** | 7.3 | 137.4 | **+** | **-** | **-** |
| 7 | 63.1 | F | 50 | 3.64 | 1.76  (F2) | 1.79  (F2) | 0.49  (F0-1) | | 0.26  (F0-1) | **+** | **+** | **+** | 8.3 | 15.6 | **-** | **-** | **-** |
| 8 | 65.4 | M | 27 | 5.90 | 1.05  (F0-1) | 1.61  (F2) | 0.17  (F0-1) | | 0.75  (≥ F2) | **-** | **+** | **-** |  |  | **+** | **-** | **-** |
| 9 | 39.6 | M | 64 | 7.40 | 0.53  (F0-1) | 1.73  (F2) | 0.36  (F0-1) | | 1.17  (F4) | **+** | **+** | **-** |  |  | **+** | **-** | **-** |
| 10 | 59.6 | M | 40 | 4.90 | 1.80  (F2) | 1.81  (F2) | 0.47  (F0-1) | | 0.32  (F0-1) | **+** | **+** | **+** | 3.5 | 125.8 | **-** | **-** | **-** |
| 11 | 74.8 | F | 40 | 6.72 | 27.5  (≥ F3) | 10.4  (≥ F3) | 6.50  (F4) | | 2.2  (F4) | **+** | **+** | **-** |  |  | **-** | **-** | **-** |
| 12 | 62.8 | M | 50 | 4.93 | 3.84  (≥ F3) | 2.54  (F2) | 1.10  (F4) | | 0.59  (≥ F2) | **+** | **+** | **+** | 1.6 | 113.1 | **-** | **-** | **-** |
| 13 | 57.3 | F | 32 | 6.28 | 4.13  (≥ F3) | 8.25  (≥ F3) | 2.46  (F4) | | 3.80  (F4) | **-** | **+** | **+** | 1.0 | 102.2 | **-** | **-** | **-** |
| 14 | 56.3 | M | 72 | 7.07 | 1.13  (F0-1) | 2.38  (F2) | 0.54  (≥ F2) | | 0.46  (F0-1) | **-** | **+** | **+** | 1.0 | 133.7 | **+** | **-** | **-** |
| 15 | 58.0 | M | 45 | 7.17 | 2.61  (F2) | 3.29  (F3) | 0.75  (≥ F2) | | 0.57  (≥ F2) | **-** | **+** | **+** | 0.8 | 12.4 | **-** | **-** | **-** |

Abbreviations: ALT, alanine aminotransferase; APRI score, AST to Platelet Ratio Index score; FIB-4 score, Fibrosis index based on the 4 factors (age, platelet counts, ALT, AST) score; HBV, hepatitis B virus; HCC, hepatocellular carcinoma; SNVs, single nucleotide variants.

* No cirrhosis was diagnosed by imaging or histological study in patients with baseline fibrosis stage ≥ F3

**References**

1. Liu WC, Wu IC, Lee YC, Lin CP, Cheng JH, Lin YJ, et al. Hepatocellular carcinoma-associated single-nucleotide variants and deletions identified by the use of genome-wide high-throughput analysis of hepatitis B virus. J Pathol. 2017;243(2):176-92.

2. European Association for the Study of the Liver. Electronic address eee, European Association for the Study of the L. EASL Clinical Practice Guidelines: Management of hepatocellular carcinoma. J Hepatol. 2018;69(1):182-236.

3. Teng W, Chang TT, Yang HI, Peng CY, Su CW, Su TH, et al. Risk scores to predict HCC and the benefits of antiviral therapy for CHB patients in gray zone of treatment guidelines. Hepatol Int. 2021;15(6):1421-30.

4. Wu JC, Huang YH, Chau GY, Su CW, Lai CR, Lee PC, et al. Risk factors for early and late recurrence in hepatitis B-related hepatocellular carcinoma. J Hepatol. 2009;51(5):890-7.

5. Shih HH, Jeng KS, Syu WJ, Huang YH, Su CW, Peng WL, et al. Hepatitis B surface antigen levels and sequences of natural hepatitis B virus variants influence the assembly and secretion of hepatitis d virus. J Virol. 2008;82(5):2250-64.

6. Liang YJ, Teng W, Chen CL, Sun CP, Teng RD, Huang YH, et al. Clinical Implications of HBV PreS/S Mutations and the Effects of PreS2 Deletion on Mitochondria, Liver Fibrosis, and Cancer Development. Hepatology. 2021;74(2):641-55.
